# Malaria in Burkina Faso: A comprehensive analysis of spatiotemporal distribution of incidence and environmental drivers, and implications for control strategies

**DOI:** 10.1371/journal.pone.0290233

**Published:** 2023-09-13

**Authors:** Cédric Bationo, Mady Cissoko, Abdoulaye Katilé, Bry Sylla, Ambroise Ouédraogo, Jean Baptiste Ouedraogo, Gauthier Tougri, Sidzabda C. B. Kompaoré, Nicolas Moiroux, Jean Gaudart

**Affiliations:** 1 Aix Marseille Univ, INSERM, IRD, ISSPAM, SESSTIM, UMR1252, Marseille, France; 2 MIVEGEC, Univ. Montpellier, CNRS, IRD, Montpellier, France; 3 Malaria Research and Training Center—Ogobara, Doumbo (MRTC-OD), FMOS-FAPH, Mali-NIAID-ICER, Université des Sciences, des Techniques et des Technologies de Bamako Mali, Bamako, Mali; 4 Direction des Systèmes d’Information en Santé, Ministère de la Santé du Burkina Faso, Ouagadougou, Burkina Faso; 5 Programme National de Lutte contre le Paludisme, Ministère de la Santé du Burkina Faso, Ouagadougou, Burkina Faso; 6 Institut de Recherche en Sciences de la Santé (IRSS), Bobo Dioulasso, Burkina Faso; 7 Aix Marseille Univ, INSERM, IRD, ISSPAM, SESSTIM, UMR1252, APHM, Hop Timone, BioSTIC, Biostatistic & ICT, Marseille, France; Clinton Health Access Initiative, UNITED STATES

## Abstract

**Background:**

The number of malaria cases worldwide has increased, with over 241 million cases and 69,000 more deaths in 2020 compared to 2019. Burkina Faso recorded over 11 million malaria cases in 2020, resulting in nearly 4,000 deaths. The overall incidence of malaria in Burkina Faso has been steadily increasing since 2016. This study investigates the spatiotemporal pattern and environmental and meteorological determinants of malaria incidence in Burkina Faso.

**Methods:**

We described the temporal dynamics of malaria cases by detecting the transmission periods and the evolution trend from 2013 to 2018. We detected hotspots using spatial scan statistics. We assessed different environmental zones through a hierarchical clustering and analyzed the environmental and climatic data to identify their association with malaria incidence at the national and at the district’s levels through generalized additive models. We also assessed the time lag between malaria peaks onset and the rainfall at the district level. The environmental and climatic data were synthetized into indicators.

**Results:**

The study found that malaria incidence had a seasonal pattern, with high transmission occurring during the rainy seasons. We also found an increasing trend in the incidence. The highest-risk districts for malaria incidence were identified, with a significant expansion of high-risk areas from less than half of the districts in 2013–2014 to nearly 90% of the districts in 2017–2018. We identified three classes of health districts based on environmental and climatic data, with the northern, south-western, and western districts forming separate clusters. Additionally, we found that the time lag between malaria peaks onset and the rainfall at the district level varied from 7 weeks to 17 weeks with a median at 10 weeks. Environmental and climatic factors have been found to be associated with the number of cases both at global and districts levels.

**Conclusion:**

The study provides important insights into the environmental and spatiotemporal patterns of malaria in Burkina Faso by assessing the spatio temporal dynamics of Malaria cases but also linking those dynamics to the environmental and climatic factors. The findings highlight the importance of targeted control strategies to reduce the burden of malaria in high-risk areas as we found that Malaria epidemiology is complex and linked to many factors that make some regions more at risk than others.

## Introduction

According to a report from the World Health Organization (WHO), the number of malaria cases was estimated at 241 million in 2020 compared to 227 million in 2019, an increase of nearly 14 million cases. 69,000 more people died of malaria in 2020 than in 2019 (627,000 vs. 558,000) [1].

Approximately 95% of malaria cases were recorded in the WHO African Region. Burkina Faso, Cameroon, Democratic Republic of Congo, Ghana, Mali, Mozambique, Niger, Nigeria, Uganda, and United Republic of Tanzania alone contributed to almost 70% of cases and 71% of deaths globally in 2020 and in sub-Saharan Africa, the estimated number of malaria deaths increased by 12% in 2020 compared to 2019 [1]. In 2020 in Burkina Faso, more than 11 million cases of malaria were recorded in the country’s health facilities, representing about 5% of global cases, resulting in nearly 4,000 deaths, according to the WHO and the National Malaria Control Program in Burkina Faso [1, 2]. The overall incidence in Burkina Faso has been steadily increasing since 2016 [3].

WHO has updated its global malaria strategy to reflect the lessons learned over the past five years [4]. To achieve the goals of this strategy (a 90% reduction in malaria incidence and mortality by 2030), new approaches and strengthened efforts are needed, supported by new tools and improved implementation of existing tools [1, 4]. The tools recommended for prevention and intervention include geo-epidemiological studies: identification of high-risk areas and periods at fine spatial and temporal scales [5–7], consideration of environmental, meteorological and socio-demographic factors in the implementation of control and prevention interventions [8–10], detection of heterogeneity patterns in malaria endemic areas [3, 11]

Even if spatial analysis of Malaria has been used in different regions [6, 8, 12, 13], implementing a spatio-temporal approach within a national malaria program is less common [14, 15].

In Burkina Faso, few studies of this kind have been carried out at the national level, defining different zones in terms of incidence, meteorological, environmental and socioeconomic factors, which would help in the decision to implement control and prevention policies in the areas most at risk [16–20].

In this geo-epidemiological study, we assessed malaria spatio-temporal heterogeneity in all 70 health districts of Burkina Faso and over five consecutive epidemic years, updating the spatial stratification of malaria, studying the differences in incidence between these years. We also assessed the different meteorological and environmental factors associated with the malaria spatio-temporal heterogeneity.

## Materials and methods

### Study area and datasets

Burkina Faso is a West African country surrounded by Mali (north and west), Niger (east), Benin, Togo, Ghana, and Côte d’Ivoire (south). In 2020 its population was estimated to be 20,903,278 [21]. The climate is Sahelian in the northern part and Sudanese in the rest of the country. The dry and cool season occurs from November to February, during which the Harmattan, a strong wind originating from the Sahara, blows, characterized by a great thermal amplitude between day and night. From March to May, heat and dry conditions prevail. The highest rainfall period occurs in July and August. The level of rainfall goes from more than 1,300 mm in the southwest, the most productive region of the country, to less than 254 mm in the north.

The health system in Burkina Faso has three administrative levels: central, intermediate, and peripheral. The central level is composed of the central structures organized around the Minister’s office and the General Secretariat. The intermediate level includes 13 regional health directorates, and the peripheral level is made up of 70 health districts. The health district is the spatial unit of the national health system. In 2018, the median number of inhabitants of health districts was 255,000 (interquartile range: 177,331) [22].

In Burkina Faso, a case of malaria is defined as a person with fever and a positive Rapid Diagnostic Test (RDT) or thick blood microscopy diagnostic, following the WHO definition [23]. The national epidemiological surveillance includes an epidemiologic information system for priority diseases, based on the DHIS2 system, including malaria case collection on a weekly basis. The malaria case data from 2013 to 2018 was collected from the national epidemiological surveillance system, which undergoes quality checks at multiple levels and includes cross-checking with patient registers, ensuring data completeness and timeliness, with no significant issues found in our own data quality assessment.

The authors did not have access to any information that could identify individual participants during or after the data collection process.

Meteorological data were collected for each health district from remote sensing using satellites through Google earth engine [24] from January 2013 to December 2018.We collected daily precipitation data (Climate Hazards Group InfraRed Precipitation with Station Data, spatial resolution: approximately 5.56 km), average daily temperature, maximum daily temperature, and minimum daily temperature data (Latest climate reanalysis produced by ECMWF / Copernicus Climate Change Service, spatial resolution: approximately 27.78 km), 8-day normalized difference vegetation index (MODIS Terra Surface Reflectance 8-Day Global, spatial resolution: approximately 0.5 km), daily surface atmospheric pressure data (Latest climate reanalysis produced by ECMWF / Copernicus Climate Change Service, spatial resolution: approximately 27.78 km), and 8-day fire detection data (Fire Information for Resource Management System, spatial resolution: approximately 1 km). We provide a table of all the variables and their sources in S1 Table. Shapefiles of Burkina Faso Health districts were extracted from the GADM (version 3.6, Davis, CA, USA) Center for Spatial Sciences at the University of California, Davis and Open Street Map websites [25].

### Statistical methods

We used the number of weekly malaria cases and assumed a constant population within each year to estimate the overall incidence time series (2013–2018), estimating trend and seasonality. We used an additive decomposition to highlight the seasonality, using the LOESS (locally estimated scatterplot smoothing) method. LOESS regression is a non-parametric approach that uses a locally weighted regression to fit a smooth curve across the points of a scatterplot [26].

We also determined the different transmission periods through a change point analysis [27] of the mean using the Pruned Exact Linear Time (PELT) algorithm and the Modified Bayes Information Criterion (MBIC) [28–30]. The change point analysis is a method applied on a series of time-ordered data to detect whether changes have occurred, determining the number of changes and estimating dates of changes [31, 32].

We also observed the density in the incidence through the epidemic years using ridgelines [33]. Ridgelines are a type of data visualization that displays multiple overlapping probability density functions or distributions. They are used to visualize the distribution of a continuous variable across different categories or groups.

An epidemic malaria year can be defined as the 12-month period commencing with the initial rise in the incidence of cases, corresponding to the onset of the primary peak, and extending through the subsequent year. The evolution of incidence across the country through epidemic years was assessed through choropleths incidence maps at the health district level. This could possibly allow observations of trends in spatial variations over epidemics years.

We investigated high-risk districts for each epidemic year and each transmission period to document the spatial pattern of the riskiest districts. We used a Poisson model purely spatial Kulldorf scan statistic [34] implemented in rsatscan [35]. Kulldorf scan statistics were used to identify hotspots and cold spots in a territory. This approach groups different spatial units adjacent to the study area into potential clusters. It was based on an elliptical window of different size, called a scan window, moving over the study area. They determine the randomness of the distribution of a phenomenon in space [34]. This randomness is used as a statistic test whose objective is to test the presence of a cluster within a region R with the following hypotheses:

H0 = Random distribution of events in R

H1 = Existence of a cluster ʗ of events, included in R, in which the probability of occurrence of an event is greater than that in the rest of R.

In our study a district was defined as a hotspot if it belongs to one of the significant clusters detected by the Kulldorf scan statistics. We used an elliptical window, a maximum cluster size set at 30% of the population at risk and the scan statistic was tested with 9999 replications of a Monte Carlo algorithm.

As malaria distribution has been shown to depend on environmental and meteorological factors that can favor vectors development and host-vector interactions [36, 37], we then produced choropleths maps for the main meteorological and environmental data retrieved for the entire period to characterize the districts environment. For the need to have a more detailed characterization of the districts environmental profile, we implemented a principal component analysis (PCA) [38, 39] on all the environmental and meteorological data from the 70 districts for the entire study period to create Synthetic Environmental National Indicators (SENIs). The PCA step can be considered as a denoising step which can lead to a more stable clustering. We then made a hierarchical ascendant cluster analysis to create environmental profiles for the districts [40, 41]. We performed the hierarchical clustering using the Ward’s criterion [42, 43] (based on the multidimensional variance).

The association between malaria and meteorological factors is complex due to the non-linear pattern of meteorological factors with the incidence., We fitted a multivariate generalized additive model (GAM) with a negative binomial distribution and a smoothing spline function on the SMIs values to model the malaria cases as a function of the SENIs [44]. A generalized additive model (GAM) is a generalized linear model with a linear predictor involving a sum of smooth functions of covariates. The GAM can be used with nonparametric smoothing terms instead of constant parameters [44–46], with the following the structure:

$$g(\mu_{i})=X_{i}*\theta +f_{1}(X_{1i})+f_{2}(X_{2i})+\cdots$$ (1)

where *μ*_*i*_ = *E*(*Yi*) and *Yi* ~ exponential family distribution.

*Yi* is a response variable, *Xi* is a row of the model matrix for any strictly parametric model components, *θ* is the corresponding parameter vector, and the *f*_*j*_ are smooth functions of the covariates, *X*_*k*_

For estimation, the *f*_*i*_, are decomposed on a spline basis. We note *b*_*jk*_ the k^th^ function of the chosen spline basis. The smooth functions *f*_*j*_ can be written as follows:

$$f_{j}(x)=\sum_{k=1}^{l}b_{jk}(x)\beta_{jk},$$ (2)

where *β*_*jk*_ is unknown.

This allowed us to assess the non-linear relations between the SMIs and malaria cases. A first-order autoregressive correlation [47, 48] was integrated into the variance-covariance matrix to account for temporal autocorrelation of malaria cases. We used a spline smoothing function on each SENI, ƒ_j_(SENI_j_) to estimate the variation of each indicators effect on the malaria cases.

Burkina Faso having different environmental profiles (refer to clustering on environmental factors), we also estimated for each district the lag of time between malaria cases and rainfall for the entire study period. We chose rainfall because it appears to be one of the major meteorological factor affecting malaria incidence [19, 49, 50].

We used a univariate generalized additive model (GAM) with a negative binomial distribution and a smoothing spline function on the rainfall values to model the malaria cases as a function of rainfall for time lags ranging from 1 to 20 weeks. The model minimizing the generalized cross-validation (GCV) was the model highlighting the lag [46, 51, 52]. Generalized Cross-Validation (GCV) is a statistical method used for model selection and parameter estimation in various regression or smoothing techniques [51]. It is commonly used in generalized additive models (GAMs) to determine the optimal amount of smoothing or regularization to apply to the model. In GAMs, smoothing is used to capture non-linear relationships between variables and the response variable. GCV is a technique that helps determine the appropriate level of smoothing by balancing the model’s fit to the data and its complexity. The goal is to find the smoothing parameter that provides the best compromise between overfitting (capturing noise or irrelevant patterns in the data) and underfitting (oversimplifying the relationships and missing important patterns)

At this health district level, after performing another PCA this time using all the environmental, meteorological, and geographic data, building, for each of the 70 districts, Synthetic Environmental District Indicators (SEDIs) and discriminated for each epidemic year for the entire study period, we fitted a multivariate generalized additive mixed model to assess the relation between association between malaria and SEDI by district. To account for time process, a first-order autoregressive process was integrated into the variance-covariance matrix of the GAMM model. The logarithm transformation of the population for each district and for each epidemic year was used as the offset in order to compute the standardized incidence ratio.

Our analysis incorporated spatially resolved environmental variables, such as rainfall, temperature, and vegetation index, which indirectly accounted for spatial autocorrelation in our study. By including these variables in the multivariate generalized additive models (GAMs), we captured the spatial patterns and variations in factors influencing malaria transmission. These variables provided valuable information on the spatial variability of environmental conditions, which are known to influence mosquito populations and malaria transmission dynamics. Consequently, the models effectively captured the spatially dependent relationships between environmental variables and malaria incidence. While explicit modeling of spatial autocorrelation through the inclusion of health district as a random effect could have provided additional insights, our approach accounted for spatial effects by including these environmental variables. This indirect consideration of spatial autocorrelation aligns with the available data and the objectives of our study, providing valuable insights into the spatial dynamics of malaria transmission in Burkina Faso.

In the final model, we used a spline smoothing function on each SMI, ƒ_j_(SMI_j_) to estimate the nonlinear relationship between each indicator and malaria cases.

While we utilized a robust dataset for our study, it is important to acknowledge potential limitations related to the data. The primary sources of these data are passive surveillance systems, which are inherently subject to certain biases. Cases of malaria may be underreported due to asymptomatic infections or less severe cases that do not seek medical attention. Moreover, misdiagnosis and uneven access to healthcare facilities across the country could also contribute to inaccuracies. While efforts are ongoing to enhance malaria surveillance in Burkina Faso, these challenges remain. Nonetheless, given the consistency in data collection and processing with quality checks in place, we believe the dataset provides a reasonably accurate picture of the spatiotemporal distribution of malaria across the country, despite these potential biases.

## Results

**Fig 1:**
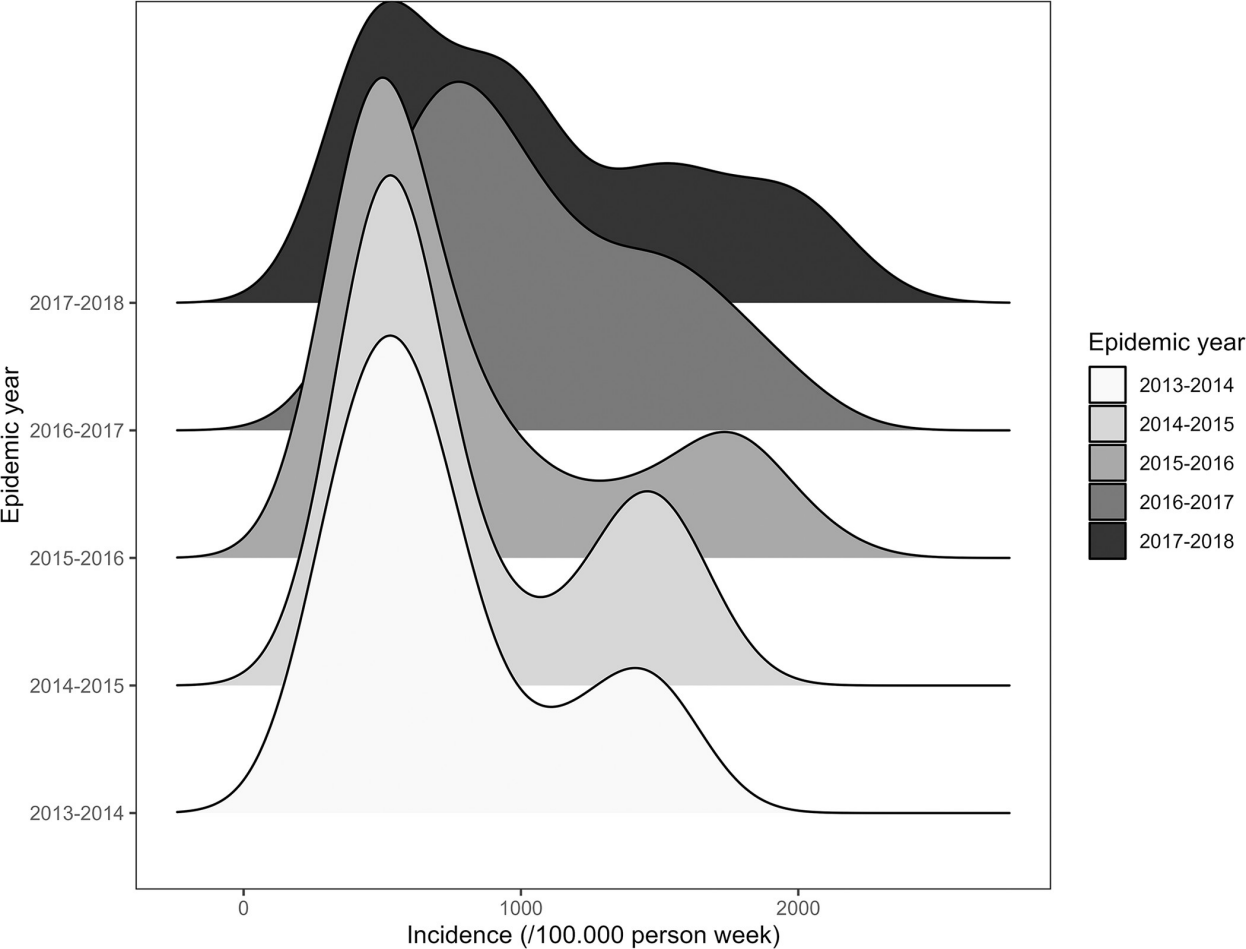
Malaria incidence rate distribution from epidemic year from 2013–2014 to 2017–2018. The X axis represents the incidence rate per 100,000-person week. The Y axis with the different shade of grey color represents the epidemics years from 2013–2014 to 2017–2018. The figure displays ridgelines to visualize the distribution of malaria incidence rates for each epidemic year. Each ridge (the shaded area) represents the distribution of incidence rates for a specific epidemic year.

The national malaria control program recorded 55,417,532 malaria cases from January 2013 to December 2018 for a population that increased from 17,322,796 in 2013 to 20,244,079 in 2018 [53]. The median malaria incidence was 739.06 cases per 100,000 population/week over the entire period (range 294.46;2427.85). The highest incidences for the years 2013 to 2015 were observed between late July and early November. During the years 2016 to 2018, high incidences were observed between mid-June and early November. In addition to that from epidemic year 2013–2014 to 2016–2017, we observed two peaks in the incidence rate distribution: the first and high one around 500 cases per 100,000 inhabitants/week and the second around 1500 cases per 100,000 inhabitants/week (Fig 1).

**Fig 2:**
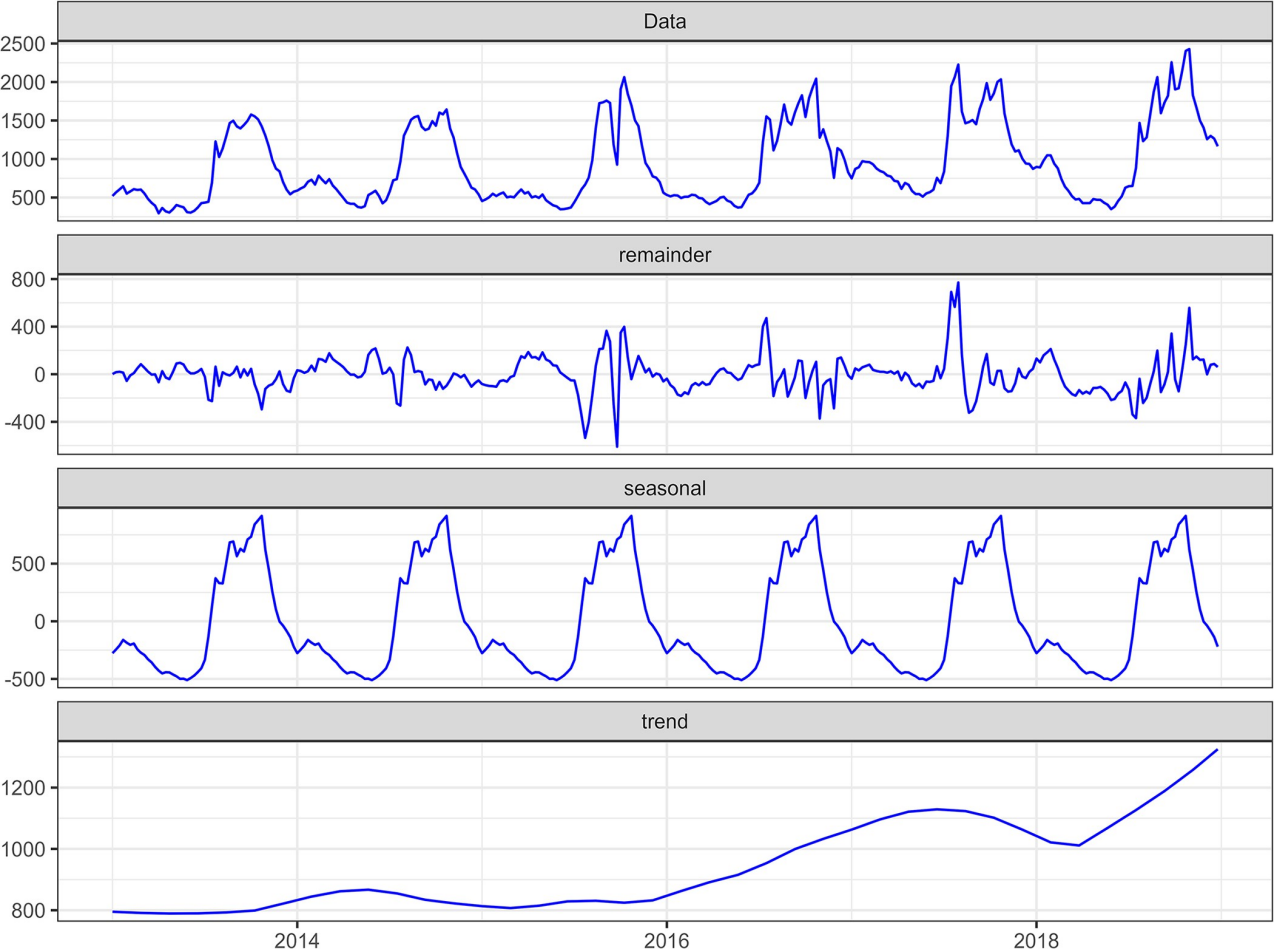
Time series of malaria incidence in Burkina Faso and its seasonal and trend components. Additive decomposition was used, and the decomposed curves were smoothed using the locally estimated scatterplot smoothing method. Raw time series (Data panel). Time series noise (Remainder panel). Time series seasons (Seasonal panel). Time series trend (Trend panel).

The decomposition in trend and season exhibited two clear phases in the time series: a stable phase with a constant incidence (around 800 cases / 100,000 person-years) from 2013 to 2015, followed by an increase phase following a nearly linear trend from 2016 to 2018 (Fig 2).

A more detailed analysis of the time series using the Change point method allowed us to characterize distinct seasonal dynamics in these two phases. The stable phase 1 (2013–2016) exhibited only two alternating transmission periods: low from January to July (included) and high from August to December (included).

**Fig 3:**
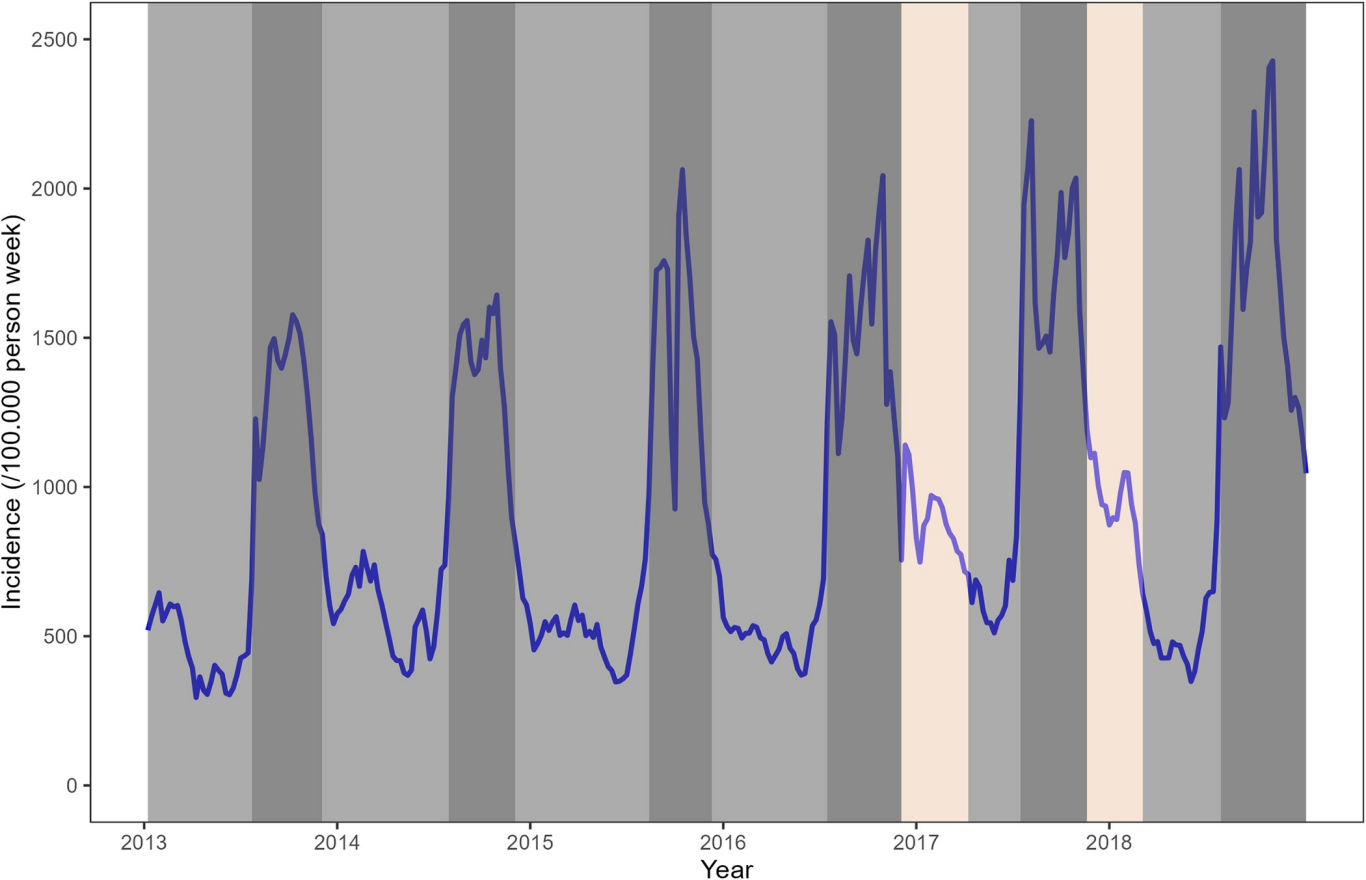
Transmission periods from 2013 to 2018, defined by the change point analysis. Low transmission period (Light grey area); High transmission period (Dark grey area); Intermediate transmission period (Tan area).

During increasing phase 2, in 2017 to 2018, an intermediate transmission period from mid-November to early January intercalated between the low transmission period from mid-February to early June and the high transmission period from July to late December (Fig 3).

Differences tests between transmission periods are presented in S1 Fig.

**Fig 4:**
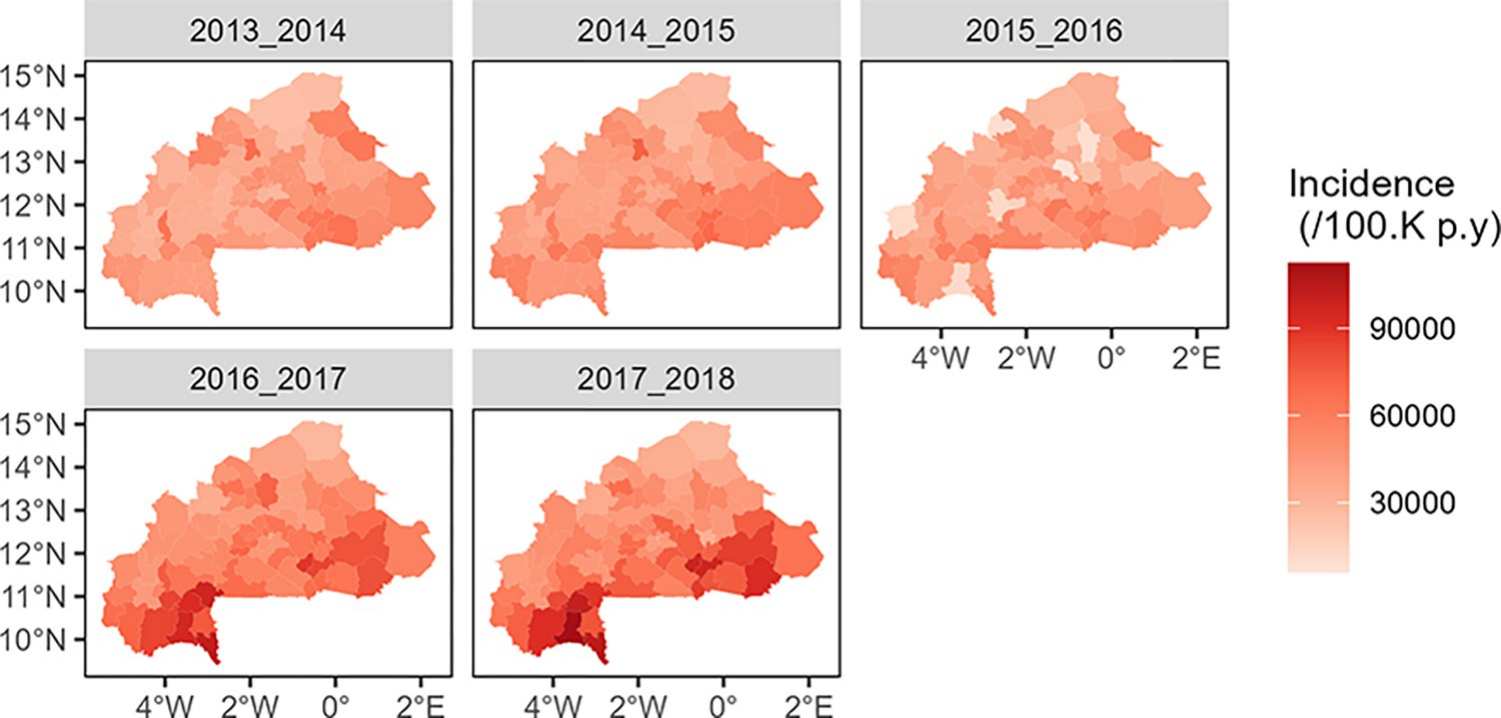
Incidence of malaria by health district, from 2013–2014 to 2017–2018; epidemic year incidence per 100,000 person/year in Burkina Faso.

**Fig 5:**
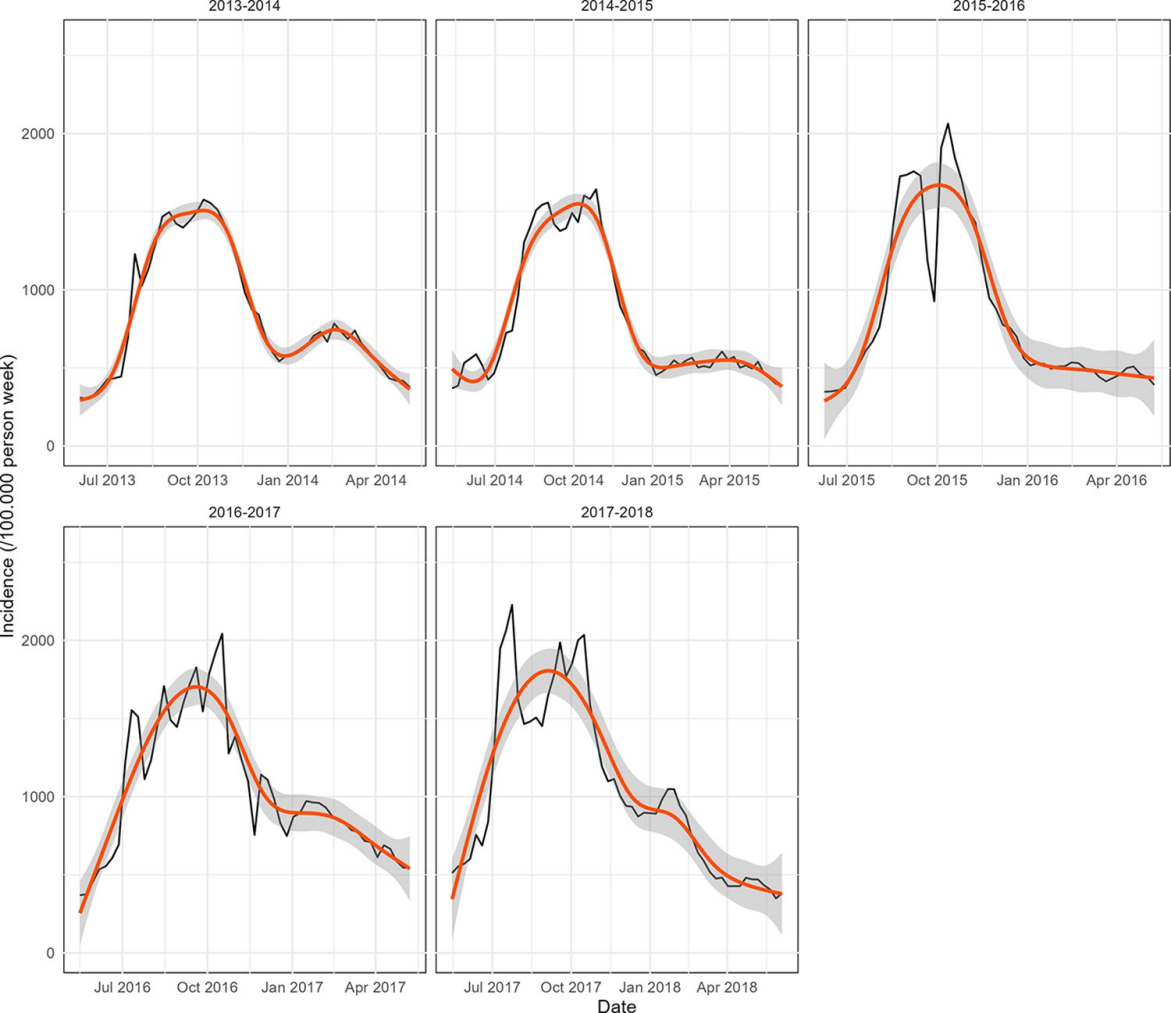
Overall trends in malaria incidence by epidemic year, from 2013–2014 to 2017–2018; weekly epidemic year incidence per 100,000 person/year with generalized additive model smoothing: Weekly incidence time series (black line) and smoothed time series (orange lines).

Choropleth maps were used to visualize the distribution and evolution of malaria incidence across the country. The analysis revealed that the health districts most affected by malaria varied from year to year. From 2013–2014 to 2015–2016, the incidence distribution was relatively homogeneous, with some districts in the center, central-eastern, and southwest regions showing higher incidence rates of at least 70,000 cases per 100,000 persons/year (see Burkina Faso region level map in S5 Fig). However, starting from the epidemic year 2016–2017, all districts, except those in the central-western region and some districts in the Sahel and central-eastern regions, experienced an incidence of at least 45,000 cases per 100,000 persons/year. The southwest, south, central-east, and east regions exhibited a particularly pronounced incidence, with rates of at least 65,000 cases per 100,000 persons/year. Notably, some districts in the southwest and east even surpassed 100,000 cases per 100,000 persons/year. Conversely, the Sahel and central-western regions consistently had lower incidence rates compared to other regions. The choropleth maps, along with the overall incidence time series analysis (Figs 4 and 5), clearly depict the increasing trend from 2016–2017 onwards. These findings provide valuable insights into the geographical distribution and temporal dynamics of malaria incidence in Burkina Faso over the study period from 2013–2014 to 2017–2018.

**Fig 6:**
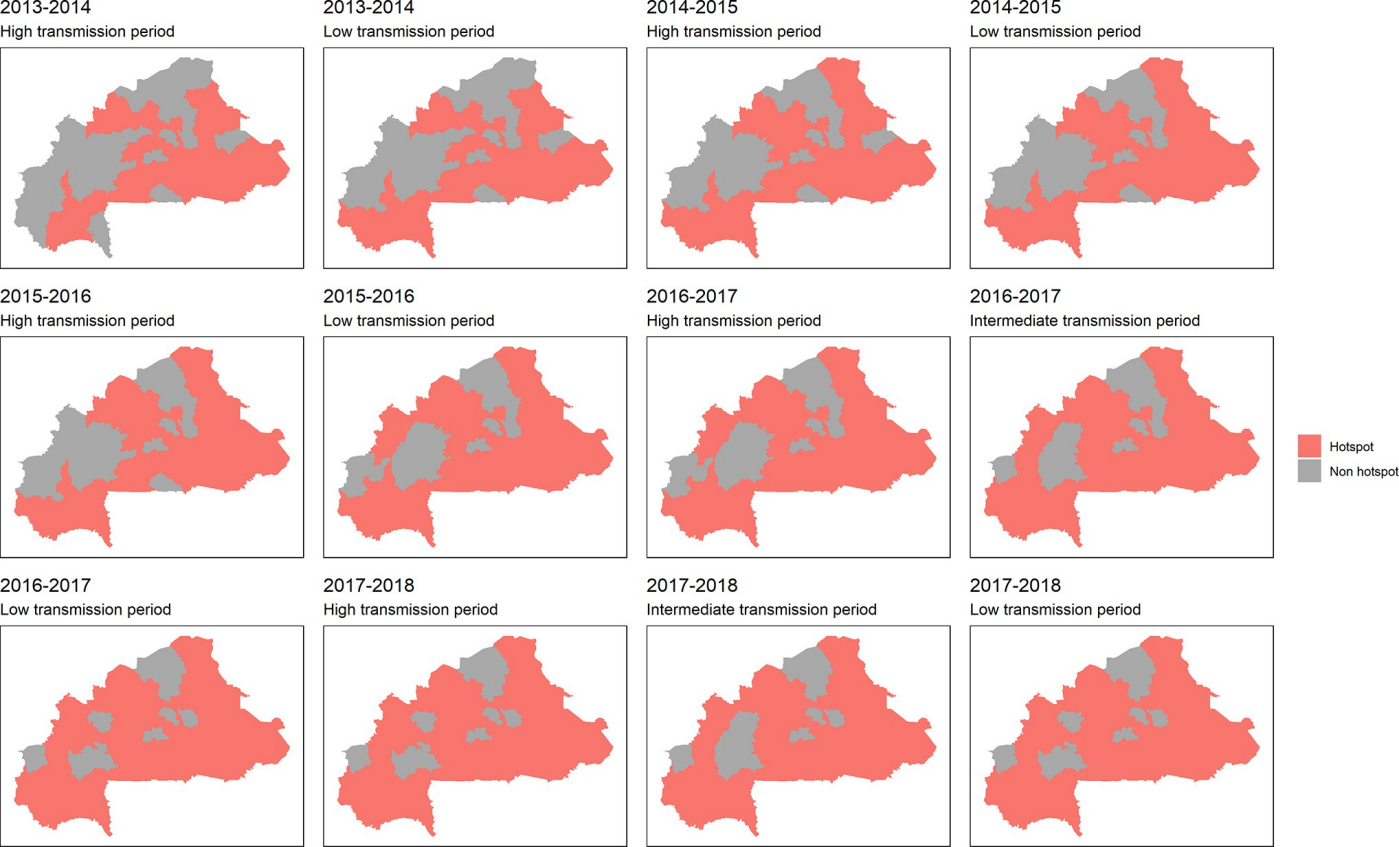
High risk health districts hotspots for each epidemic year and transmission period. A district is defined as a hotspot if it belongs to one of the significant clusters detected by the Kulldorf scan statistics.

By examining high-risk districts for each epidemic year and transmission period (by using the Kulldorf approach), we were able to identify areas with the highest risk. Fig 6 depicts the progression of districts at high risk for Malaria, demonstrating an expansion of the affected areas from less than half of the districts being at high risk in 2013–2014 to nearly 90% of the districts being at high risk in 2017–2018.

**Fig 7:**
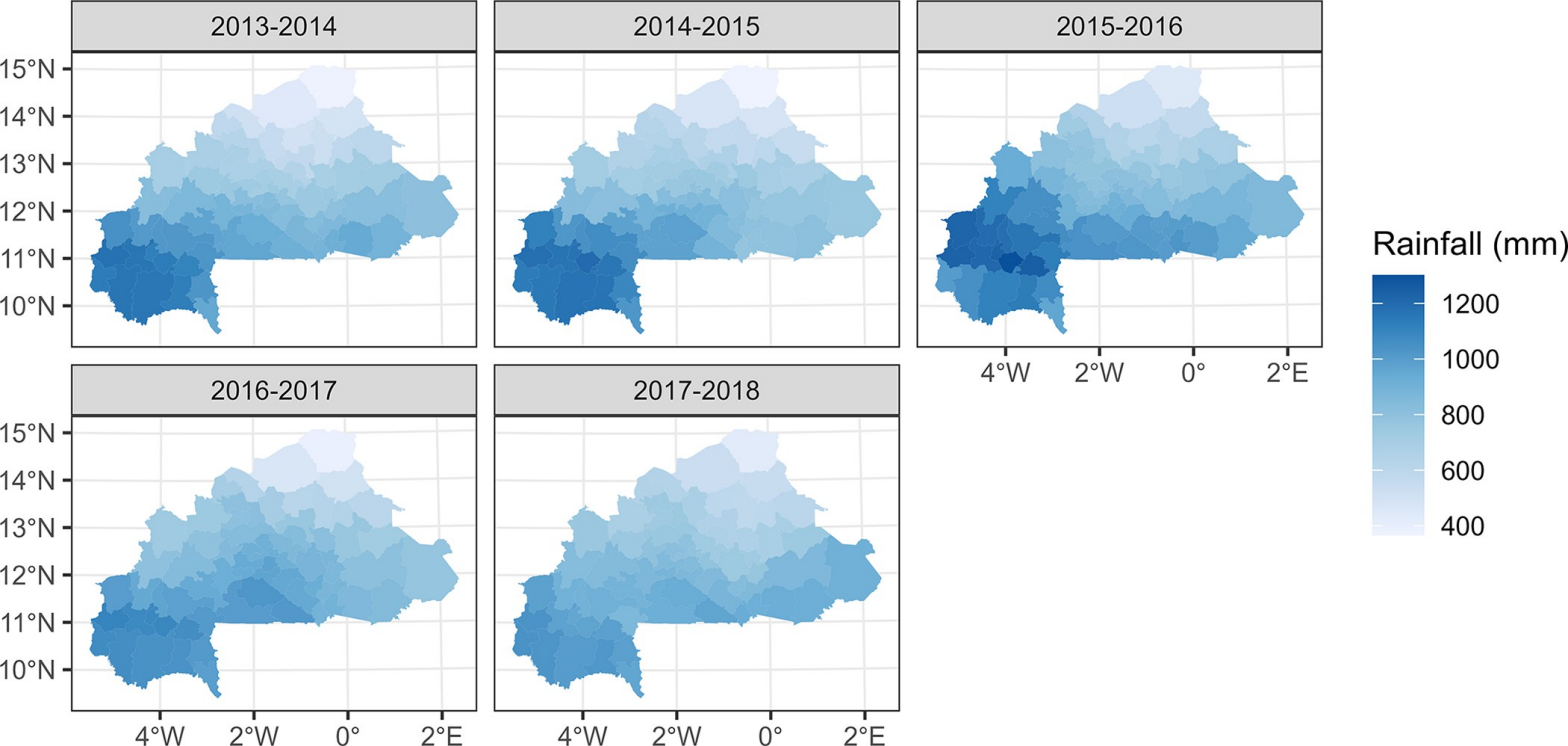
Mean rainfall (mm) maps by health district, from epidemic year 2013–2014 to 2017–2018.

**Fig 8:**
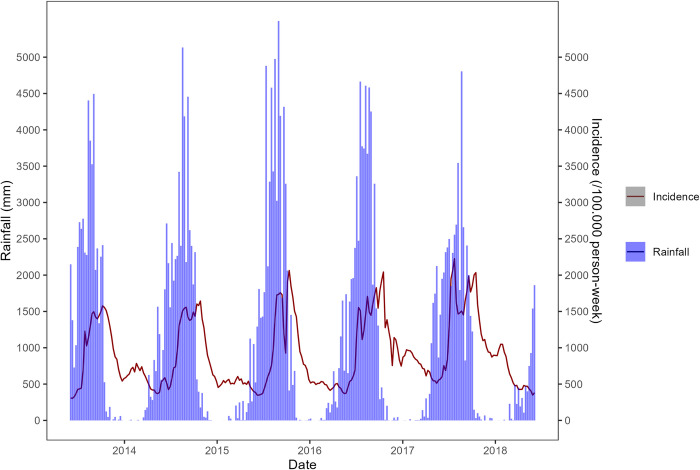
Temporal trends (time series) of mean rainfall (mm) from 2013 to 2018 (Blue bars) and malaria incidence time series from 2013 to 2018 (red line).

**Fig 9:**
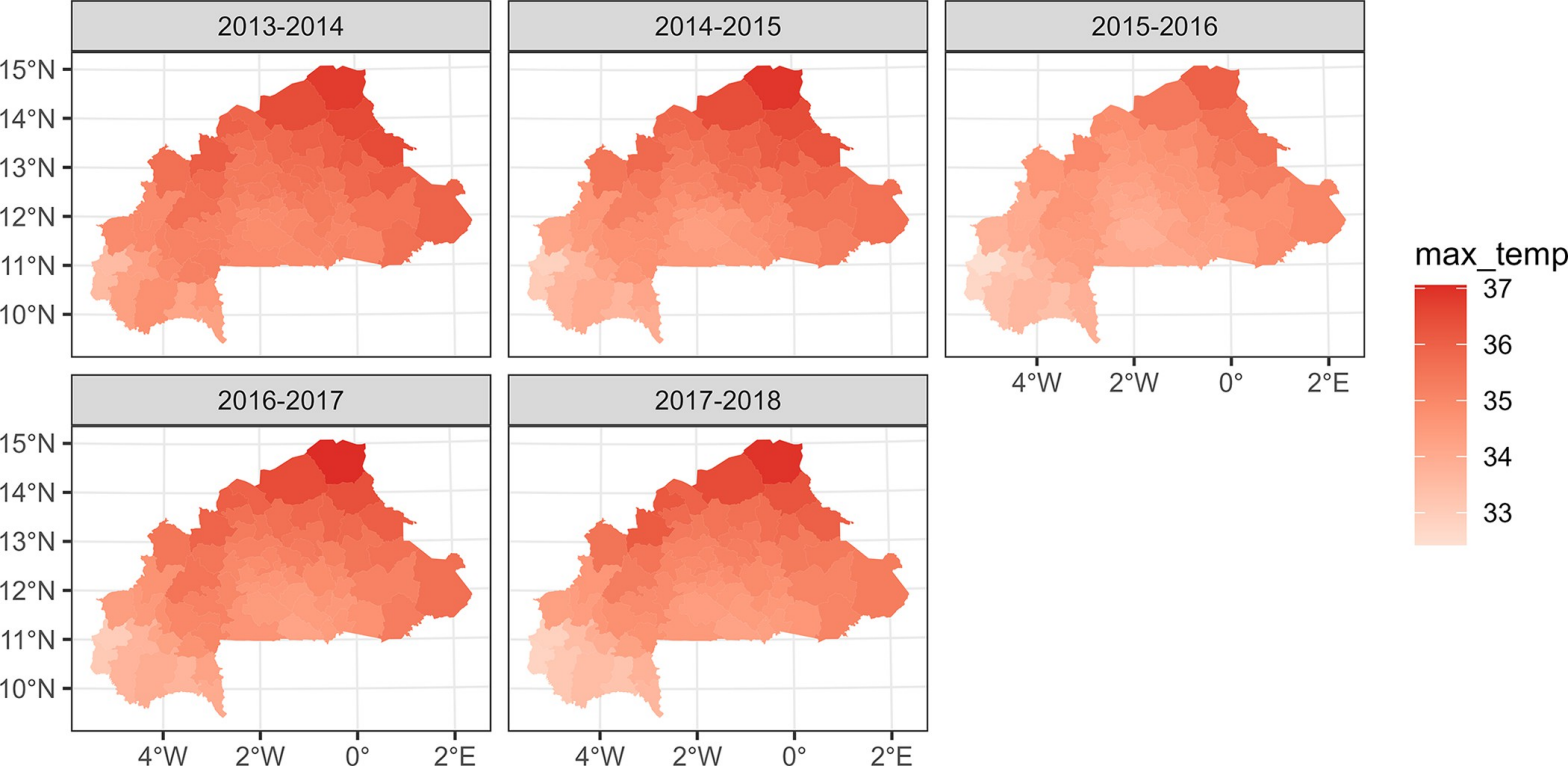
Maximum temperatures (°C) maps by health district, from epidemic year 2013–2014 to 2017–2018.

**Fig 10:**
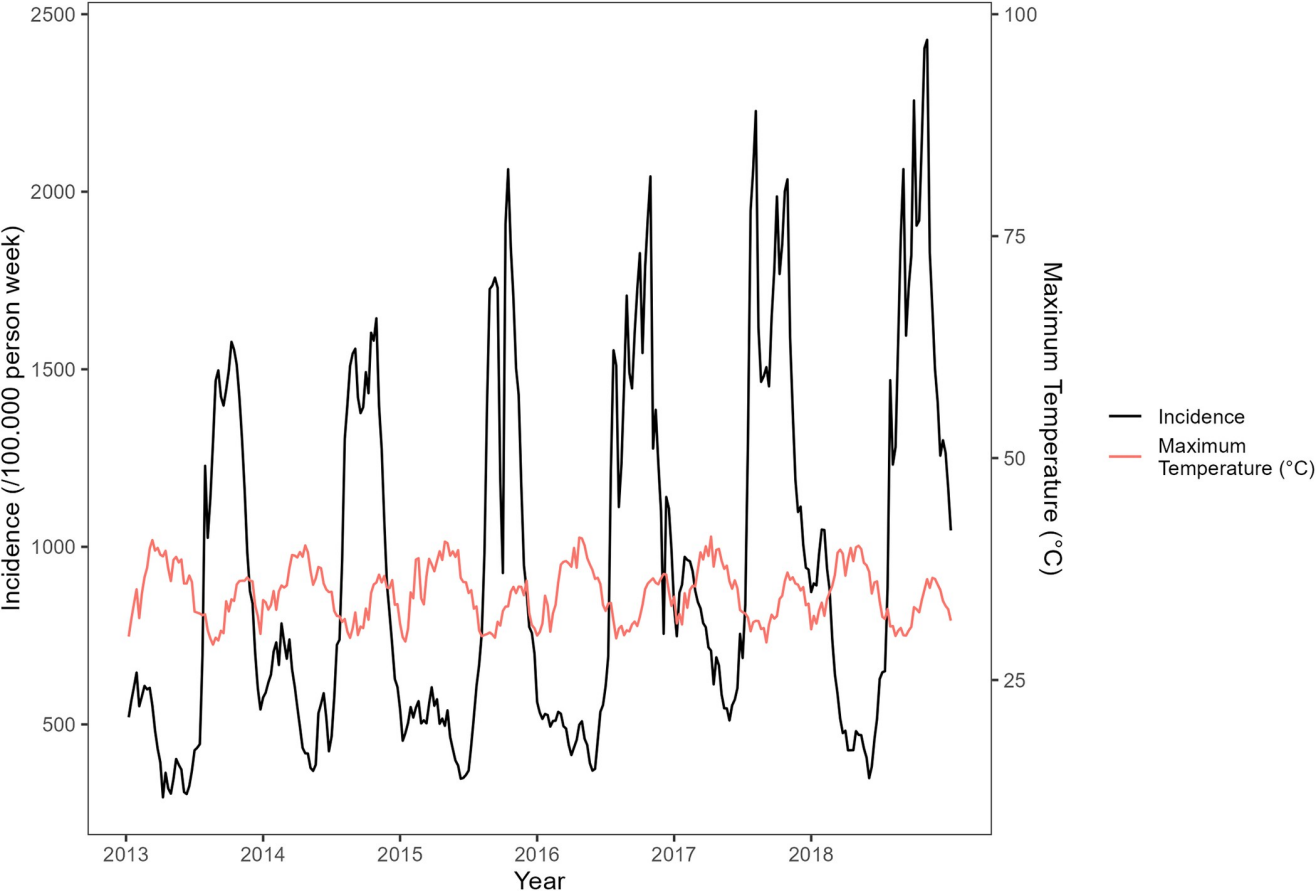
Temporal trends (time series) in maximum temperatures (°C) from 2013to 2018 (red line) and malaria incidence time series from 2013 to 2018 (black line).

**Fig 11:**
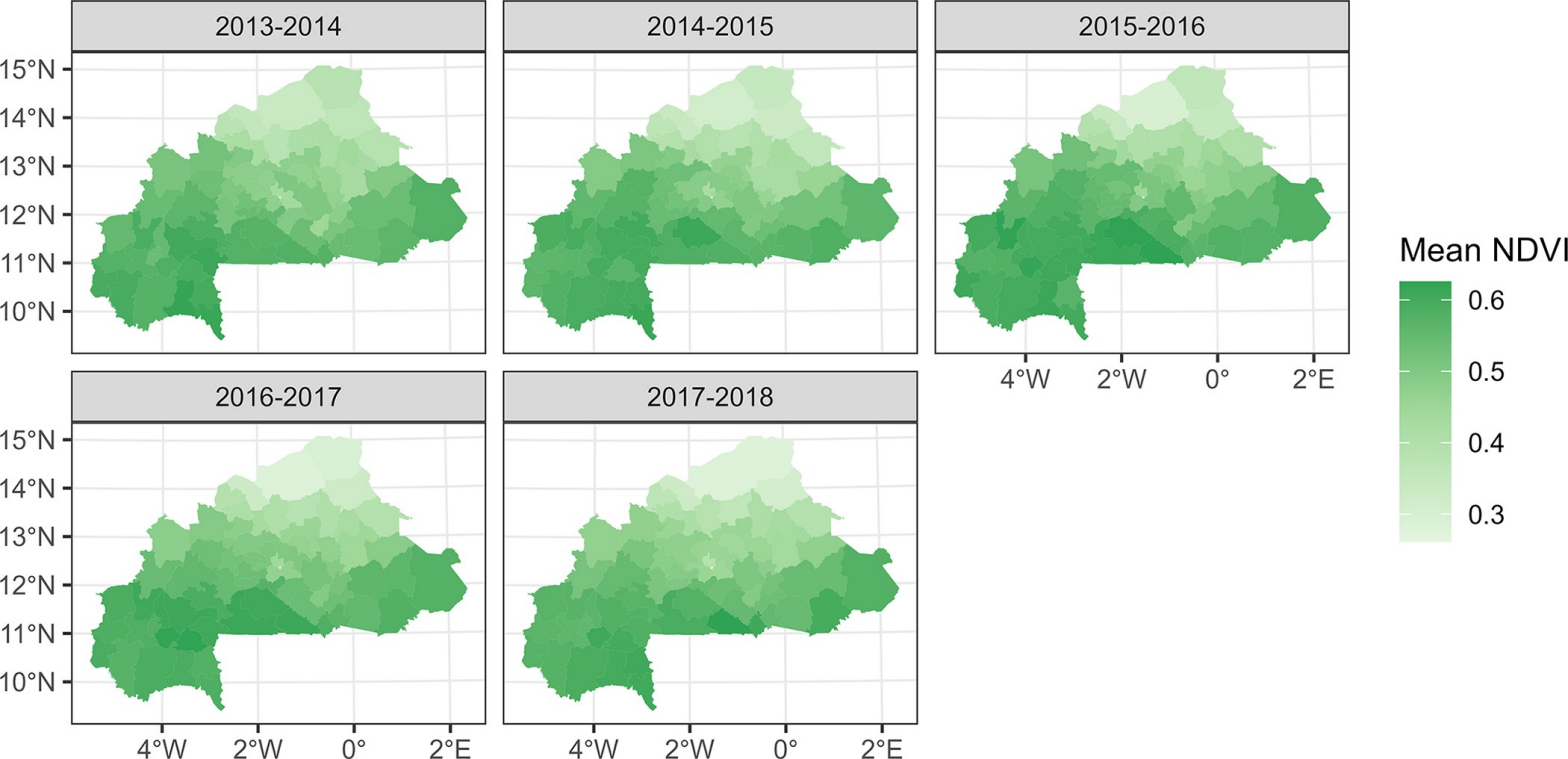
Mean NDVI maps by health district, from epidemic year 2013–2014 to 2017–2018.

**Fig 12:**
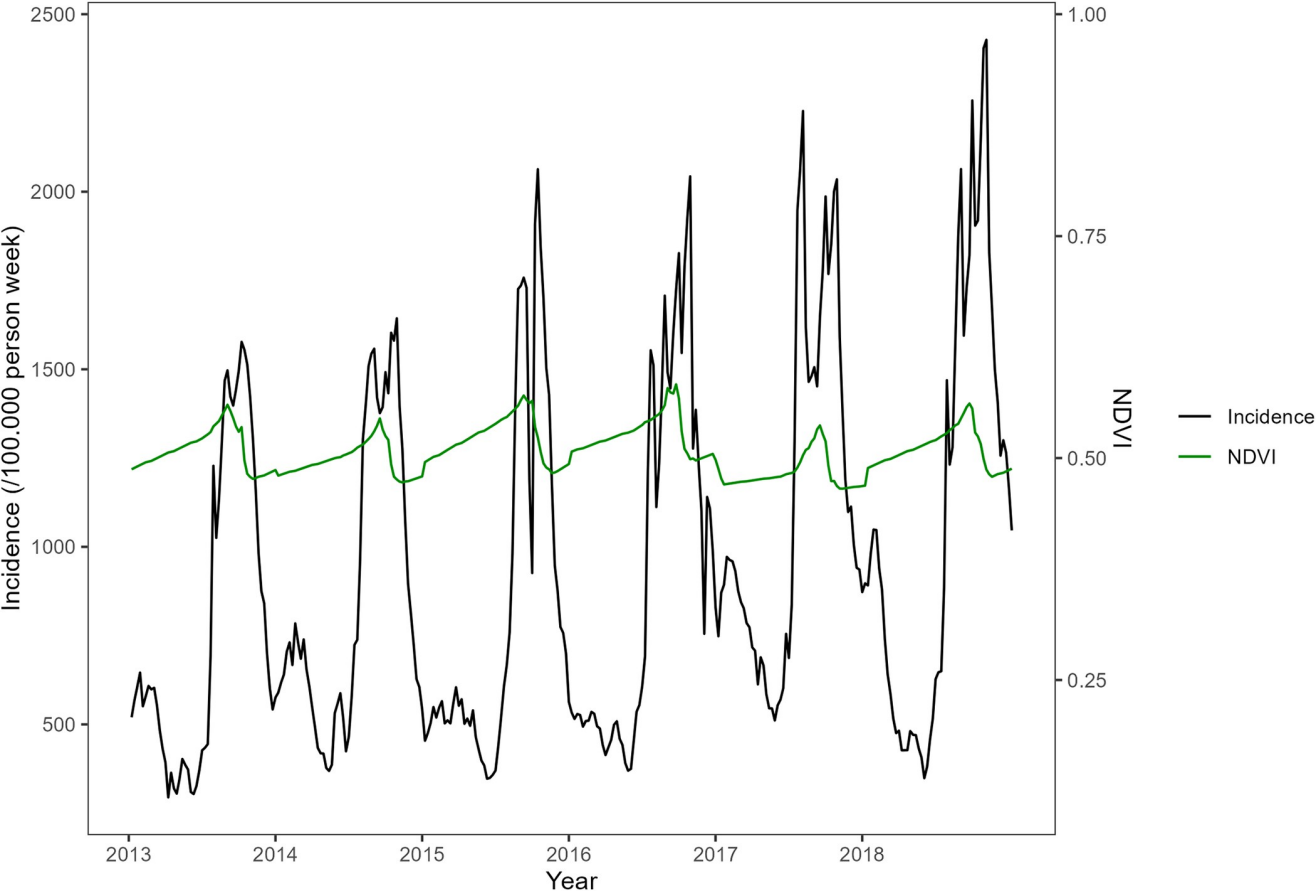
Temporal trends (time series) in NDVI from 2013to 2018 (black line) and malaria incidence time series from 2013 to 2018 (red line).

Maps of the most important environmental variables affecting malaria (mean rainfall, temperature, and normalized difference vegetation index) data over the entire study period (from the national level PCA) along with the overall time series from 2013 to 2018 are shown in Figs 7–12. Maps for the other environmental variables can be found in S2–S4 Figs For the rainfall and the NDVI data, we observed a north-south gradient with the highest rainfall and NDVI in south-west, south, central-eastern and southeastern regions. Maximum temperatures showed an opposite pattern with the lowest observed in the south-west.

**Fig 13:**
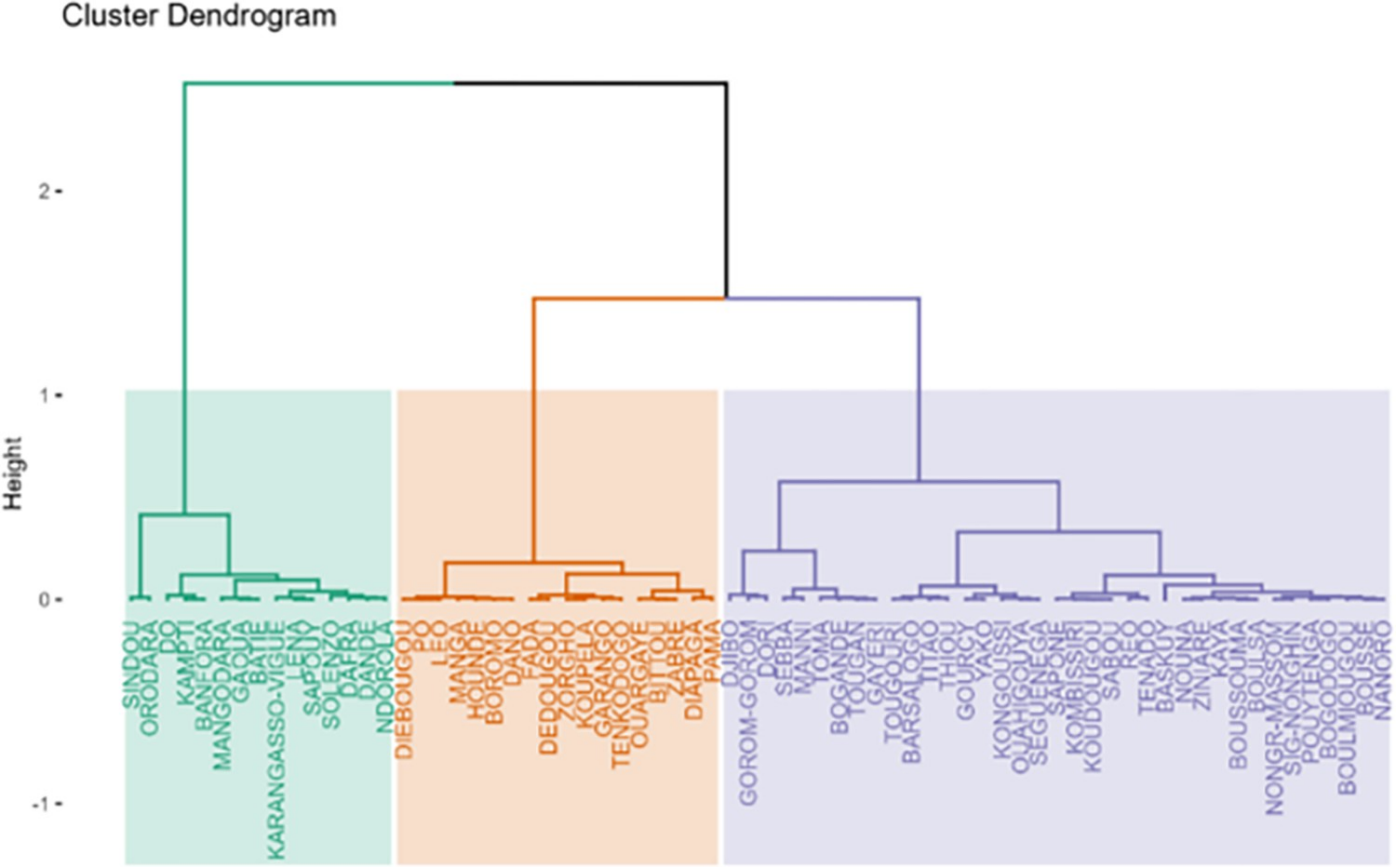
Dendrogram showing districts classification into environmental profiles. Profile 1 (Purple): High maximum temperatures, high mean temperatures, average minimum temperatures, and low rainfalls, profile 2 (Orange): High minimum temperatures, high NDVIs, high atmospheric pressures, and high medium rainfalls. and profile 3 (Green): high rainfalls, high NDVIs, and medium mean temperatures.

**Fig 14:**
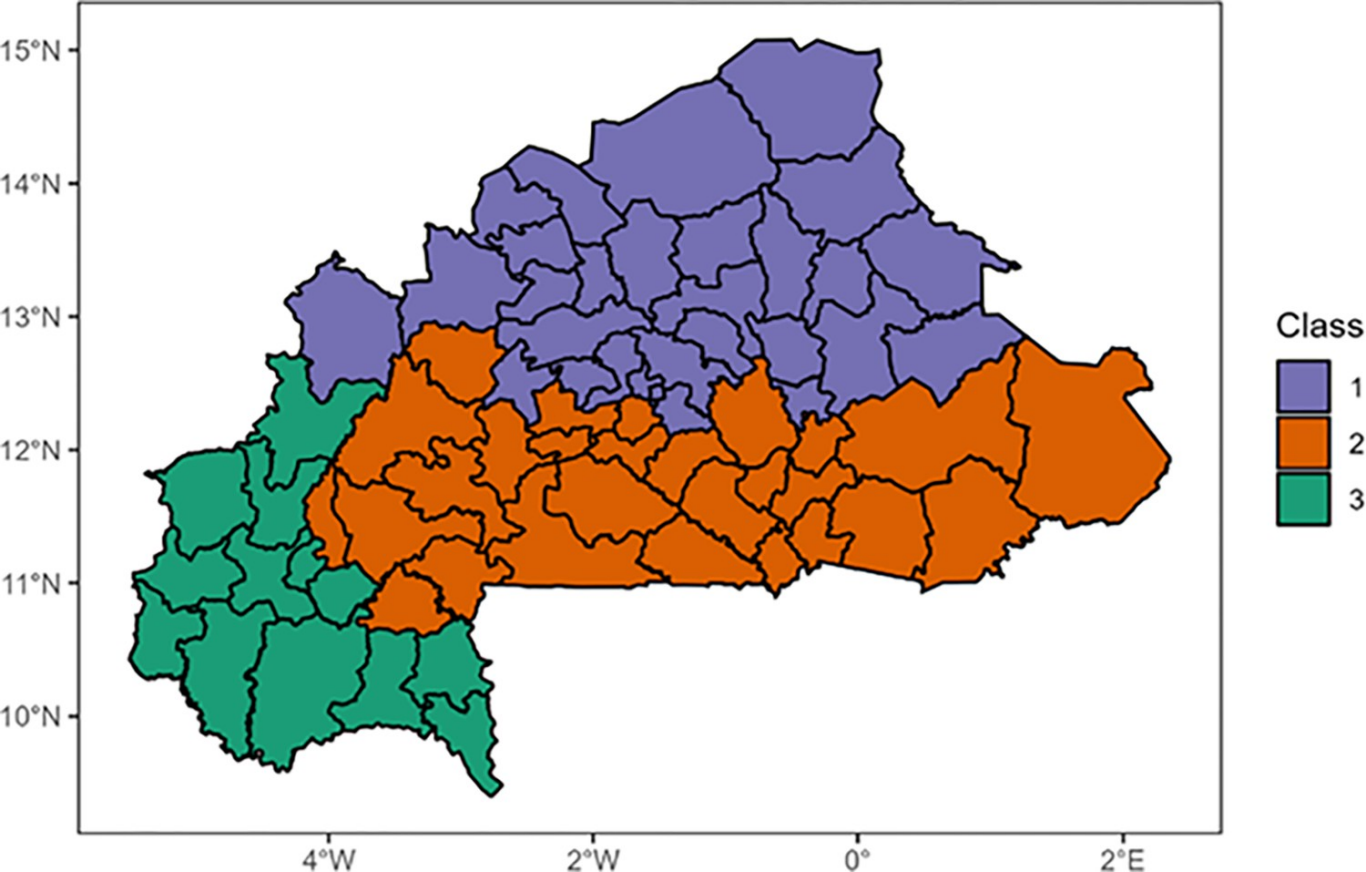
Mapping of health districts into environmental classes. Profile 1 (Purple): High maximum temperatures, high mean temperatures, average minimum temperatures, and low rainfalls, profile 2 (Orange): High minimum temperatures, high NDVIs, high atmospheric pressures, and high medium rainfalls. and profile 3 (Green): high rainfalls, high NDVIs, and medium mean temperatures.

Using the hierarchical ascendant clustering on the PCA, we detected three profiles of health districts characterized by the environmental and climatic data (Figs 13 and 14).

High maximum temperatures, high mean temperatures, average minimum temperatures, and low rainfalls were most significantly associated with the profile 1. The profile 2 was characterized by high minimum temperatures, high NDVIs, high atmospheric pressures, and high medium rainfalls. The profile 3 was characterized by high rainfalls, high NDVIs, and medium mean temperatures.

The mapping of the districts according to those profiles (Fig 14) showed a clear pattern with the districts located in north part of the country forming a cluster, those located in the south-west and the west forming another one and those located in the center to the east forming the last one.

Overall, we assessed two SENIs. The SENI1 was composed of the weekly sum of daily rainfalls, minimum temperature and NDVI. The SENI2 was composed of the maximum temperature, mean temperature, and pressure.

**Fig 15:**
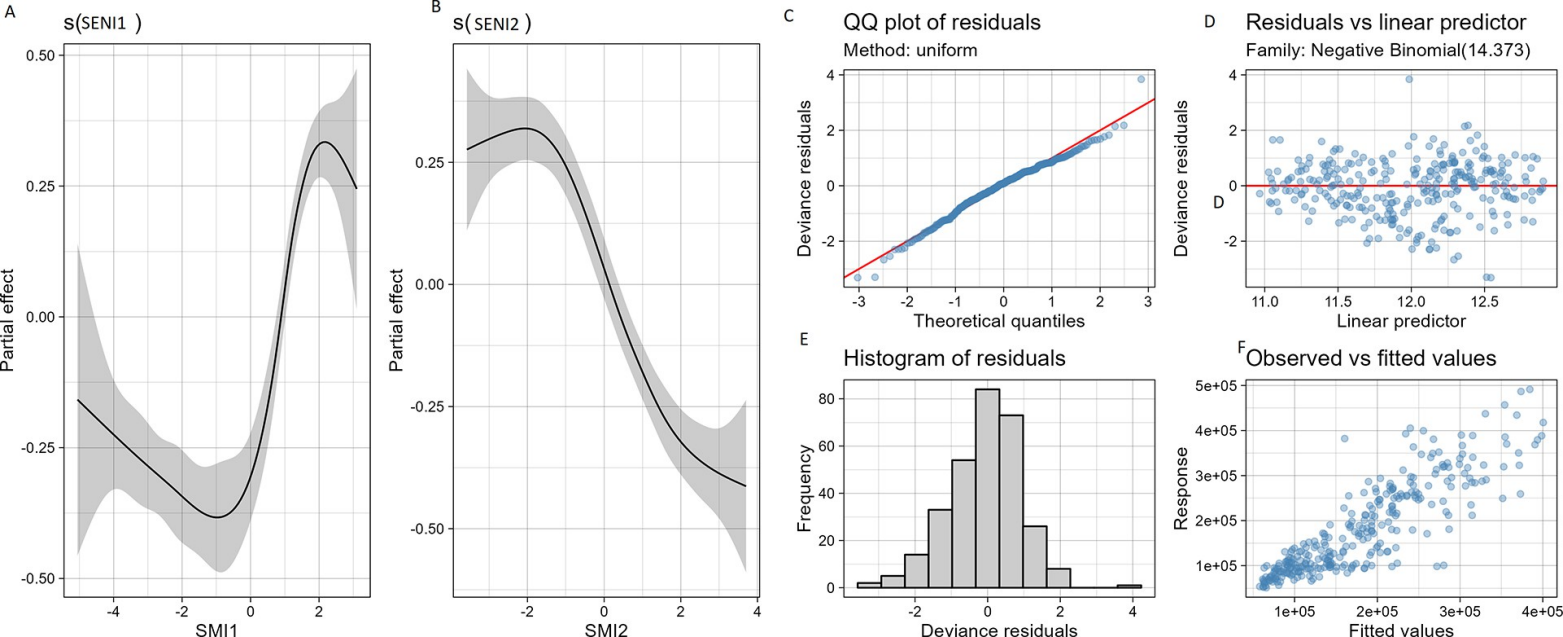
Relation between malaria cases and SENIs. Panel A: Relation between malaria global cases and SENI1 (mostly rainfall and mean temperature);. Panel B: Relation between malaria global cases and SENI2 (mostly temperature); Panel C, D,E and F: Diagnostic plot of multivariate generalized additive model.

In the multivariate GAM model (that reached 80% of deviance explained of), non-linear, significant relationships were observed at the national scale between both SENI1 and SENI2 and the number of cases (p<0.001 and p<0.001, respectively). For SENI1 (rainfall, minimum temperature, NDVI), a low asymptote was observed for almost all the lowest values and then an increasing trend for almost all the highest values reaching an asymptote at the very end (Fig 15 panel A). For the SENI2 (temperature) a nonlinear relationship was also assessed with a high asymptote for the lowest values and then a decreasing trend for almost all the values of the SENI2 (Fig 15 panel B). The graphical quality assessment of this multivariate model diagnostic showed a good confidence (Fig 15 Panel C).

**Fig 16:**
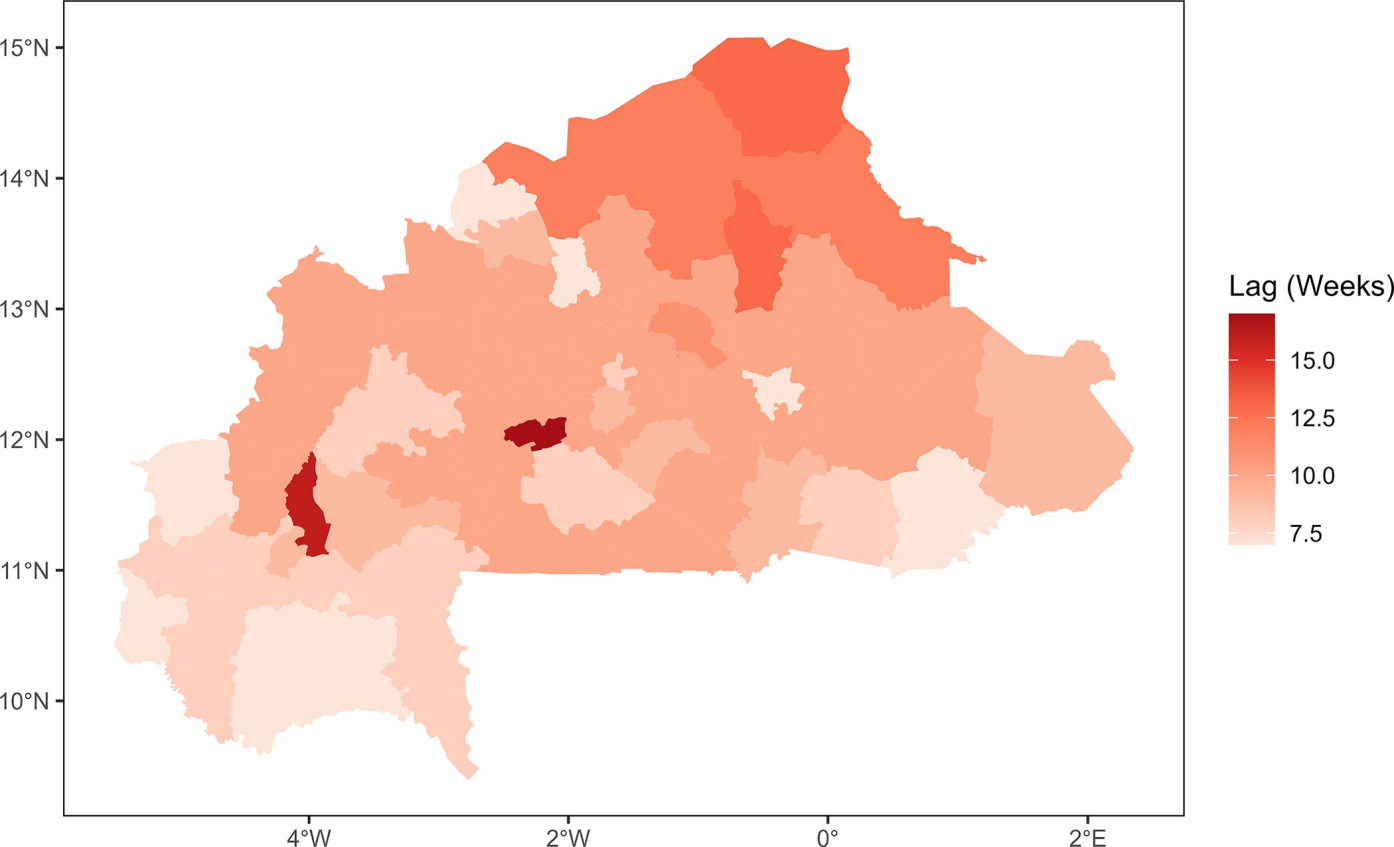
Time lags (in weeks) between cases and rainfalls at districts level. The lags had Minimum of 7 weeks, a maximum of 17 weeks and a median of 10 weeks.

The time lags between rainfalls and malaria cases, as analyzed by a univariate generalized additive model at the district level were between seven and 17 with a median of 10. Fig 16 showed a south-north gradient with the highest lag located in the north and the lowest lag in the south. However, we observed two districts located in the center and the south-west with respectively 17 weeks and 16 weeks as time lags.

From the district level PCA, we assessed the effect of three district-level Synthetic Environmental District Indicators (SEDIs): SEDI1 (Maximum temperature, mean temperature), SEDI2 (minimum temperature, NDVI and latitude), SEDI3 (Rainfall and longitude).

**Fig 17:**
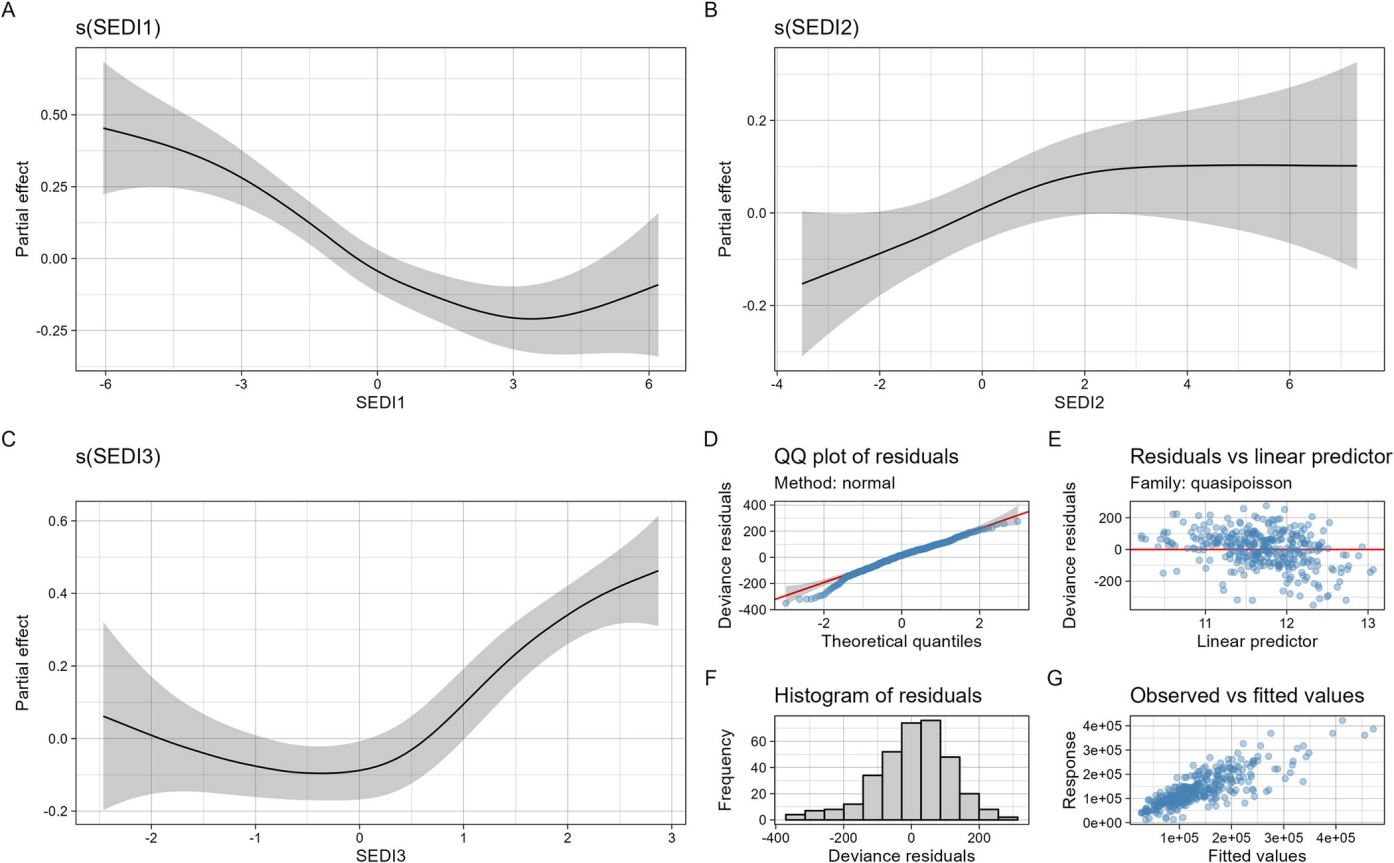
Relation between malaria cases and SEDIs at district level; Panel A: Relation between malaria global cases and SEDI1 (mostly maximum temperature); Panel B: Relation between malaria global cases and SEDI2 (mostly min temperature vegetation and latitude). Panel C: Relation between malaria global cases and SEDI3 (rainfall and longitude). Panel D,E,F and G:Diagnostic plot of multivariate generalized additive model.

In the district level multivariate model, SEDI1, SEDI2 and SEDI3 were all significantly associated with the number of cases at the district level (p<0.001, p<0.01 and p<0.01 respectively). A nonlinear relationship between the cases and the SEDI1 (maximum temperature) was observed. For SEDI1, cases were low at higher values (Fig 17, panel A). For the SEDI2 (min temperature, vegetation, and latitude) a nonlinear relationship was also assessed with an increasing trend for almost all the lowest values of the SEDI2, then reaching an asymptote for the highest values (Fig 17 panel B). For the SEDI3 (rainfall and longitude) a nonlinear relationship was assessed, beginning with an asymptote for the lowest values of the SEDI3 and then a rough increasing trend for the highest values of the SEDI3 (Fig 17, panel C). The graphical quality assessment of this multivariate model diagnostic showed good confidence (Fig 17 Panel D,E,F and G).

## Discussion

Despite the significant efforts made by the National Malaria Control Program in Burkina Faso, the incidence rate of malaria remains high every year, with an intra-annual and an inter-annual variability in the incidence. Indeed, in addition to the intra-annual seasonal dynamic of malaria, an increasing inter-annual trend in incidence was showed throughout the years from 2013 to 2018. Note that, similarly to other authors [54, 55], we observed that a complete malaria epidemic overlaps two calendar years (Fig 4). This has important implications for policy makers and public health officials. Since the malaria epidemic spans across two years, it is crucial to consider this when estimating the number of cases, and planning for prevention and control strategies, as well as funding allocations. The inter-annual increasing trend highlighted by our study reinforces the need of considering long time series and spatial distribution through time in order to better tailor malaria control strategies. This requires careful planning and coordination of resources, including funding, personnel, and supplies, to ensure that prevention and control measures are implemented effectively and consistently over the entire epidemic period. Additionally, policies and funding should be designed to ensure that there is no gap in interventions or resources as one-year ends and the next begins.

One particular feature of malaria transmission in Burkina Faso was the observed phenomenon of rebound in the incidence each year (second peak). During these periods, children were no longer protected by the Seasonal Malaria Chemoprevention (SMC), ending in October, which makes them more vulnerable to malaria infection. These results demonstrate the urgency of implementing more effective interventions to combat malaria in Burkina Faso. One potential option could be the introduction of supplementary rounds of SMC to provide additional protection in November. Indeed, SMC is shown to be effective in reducing malaria incidence in children is seasonal malaria chemoprevention (SMC) [56, 57], but the observed rebound in the incidence around November each year may expose the children to severe malaria.

In addition, malaria incidence in Burkina Faso has been found to be highly variable spatially and across different years with some regions and districts experiencing higher incidences of malaria than others. The choropleth maps provided a visualization of this variability, showing the geographical distribution of malaria incidence over time. This finding is consistent with other studies that have reported an heterogeneity in malaria incidence in Burkina Faso in recent years [3, 20].

The results presented in Fig 14 highlighted a worrisome trend of expansion of the districts at high risk for malaria in Burkina Faso over time and their sustainability during both high and low transmission period. This expansion of high-risk areas may be attributed to various factors, such as entomological factors, prevention, and control measures but also climate change, population growth, and increased human behaviors and activities leading to changes in land use and environmental conditions. The findings in Fig 14 are consistent with previous studies that suggest a rise in malaria transmission in different parts of Africa [58, 59]. One potential explanation for the observed expansion of high-risk areas is the population displacement in Burkina Faso, settling in new areas, and leading to land use changes, and a subsequent increase in the prevalence of malaria [13, 60]. Additionally, the population displacement may result in overcrowding, which may lead to poor living conditions which contribute to increased malaria transmission.

The identification of distinct profiles of health districts based on environmental and climatic data was also a noteworthy finding. The clustering analysis identified three profiles of health districts that differed in their environmental and climatic characteristics. These findings are helpful to guide targeted interventions and resource allocation to areas with the highest risk of malaria context and transmission.

The multivariate model at national level showed that both SMI1 (rainfall, minimum temperature and NDVI) and SMI2 (maximum temperature) were significantly associated with the number of malaria cases at the country level. These findings are consistent with previous studies that have shown the influence of temperature and rainfall on malaria transmission [61, 62]. The nonlinear relationship between the national cases and the SMIs suggested that the impact of environmental variables on malaria transmission may be more complex than a linear relationship. The low and high asymptotes of SMI1 and SMI2 could be due to the fact that extreme values may not be conducive for malaria transmission, while intermediate temperatures and rainfall may be optimal for mosquito survival and reproduction [61].

The district-level analysis also showed a south-north gradient in the time lag between malaria cases and rainfall, with the highest lags located in the north and the lowest lags in the south. This is consistent with previous studies that have shown a lag between rainfall and malaria incidence due to the time required for the development and maturation of mosquito eggs and larvae [63]. The longer time lags observed in the north may reflect the lower rainfall and vegetation in this region. Looking at the district level multivariate model, the overall decreasing trend of cases for dSMI1 (maximum temperature) may be explained by the fact that high temperatures can have a direct impact on the mosquito’s survival and reproductive rate, thus limiting the population size and transmission capacity of the vector[64, 65]. Extreme temperatures disrupt the synchronization of the mosquito life cycle with the seasonal rainfall patterns, leading to a reduction in the mosquito population and a less periodic transmission of malaria [64, 66, 67]. The overall increasing trend of cases for dSMI2 may be explained by the fact that mean temperature, vegetation, and latitude have been identified as important environmental factors that influence the transmission of malaria. Vegetation can impact the availability of breeding sites for mosquitoes, as well as the survival and reproductive success of the mosquito vector. Latitude can also play a role in malaria transmission, as areas closer to the equator tend to have higher transmission rates due to more humid conditions [68, 69]. For the dSMI3, the overall increase in the cases may be explained by the fact that rainfall can create suitable breeding sites. A higher amount of rainfall can lead to an increase in the number of breeding sites and therefore an increase in the mosquito population, which can result in higher transmission rates of malaria [19, 55, 70].

Overall, the findings of this study highlight the complex relationships between environmental factors and malaria transmission and suggest that targeted interventions aimed at reducing malaria transmission may need to consider a range of environmental and climatic factors at a local scale to be effective.

In our study, we employed the Generalized additive models (GAMS) for analysis; however, looking ahead, machine learning has the potential to be employed in analyzing large-scale environmental databases, providing novel insights and enhancing data analysis and decision-making processes.

Our findings, based on the analysis of malaria case data in Burkina Faso, need to be considered in light of potential biases related to the data. As previously mentioned in the methods section, issues such as underreporting, misdiagnosis, and uneven access to healthcare services can impact the accuracy of the data. These limitations are inherent in passive surveillance data and represent challenges for disease control programs globally. However, the consistent collection of data, coupled with the quality checks implemented by the National Malaria Control Program in Burkina Faso, makes it possible to derive meaningful insights from this data. Looking forward, efforts should focus on strengthening the surveillance system, improving data quality, and addressing these potential biases to further refine our understanding of the spatiotemporal dynamics of malaria. Such enhancements could lead to more accurate and effective strategies for disease control and prevention. Furthermore, while our study considered several important climate and environmental variables, there may be other factors that influence malaria transmission but were not included in our analysis. These could include socioeconomic factors, land use changes, vector control interventions, and human behavioral factors. Incorporating additional environmental factors could provide a more comprehensive understanding of the drivers of malaria transmission.

## Conclusion

This study showed the spatial and temporal distribution of malaria across the country and underscores the importance of understanding the spatiotemporal dynamics but also the environmental factors malaria transmission in Burkina Faso at a local scale to guide effective control and prevention targeted strategies. The study’s results provide important insights for policymakers and public health officials to design and implement targeted interventions that are tailored to the specific needs of different regions in the country, in line with the stratification recommended by WHO.

## Supporting information

S1 Table Meteorological and environmental variables. (DOCX)

S1 Fig Differences testing of incidence through transmission periods. Difference in the mean of the incidence through epidemics transmission periods using the Yuen’s test for trimmed means (robust *t*-test) for two groups and the empirical likelihood-based ANOVA for trimmed means test for more than two groups.
(TIF)

S2 Fig Mean pressure maps from 2013–2014 to 2017–2018. (TIF)

S3 Fig Average mean temperature maps from 2013–2014 to 2017–2018. (TIF)

S4 Fig Average minimum temperature maps from 2013–2014 to 2017–2018. (TIF)

S5 Fig Burkina Faso map with region boundaries. (TIF)

## References

[1] OMS. *Rapport sur le Paludisme dans le Monde* (2021). https://cdn.who.int/media/docs/default-source/malaria/world-malaria-reports/world-malaria-report-2021-global-briefing-kit-fre.pdf. Accessed on July 3, 2022.

[2] ministère de la Santé du Burkina Faso. *Annuaire Statistique* 2020. https://www.sante.gov.bf/fileadmin/user_upload/storages/annuaire_statistique_ms_2020_signe.pdf. Accessed on July 3, 2022.

[3] Bationo CS, Lokossou V, Landier J, Sylla B, Tougri G, Ouedraogo B, et al. Geo-epidemiology of Malaria in Burkina Faso, 2013–2018: a recent re-increase. 2021 Oct p. 2021.10.27. doi: 10.1101/2021.10.27.21265260

[4] World Health Organization. Global technical strategy for malaria 2016–2030. Available from: https://www.who.int/publications/i/item/9789240031357

[5] Landier J, Rebaudet S, Piarroux R, Gaudart J. Spatiotemporal analysis of malaria for new sustainable control strategies. BMC Med. 2018;16: 226. doi: 10.1186/s12916-018-1224-2 30509258 PMC6278049

[6] Bejon P, Williams TN, Nyundo C, Hay SI, Benz D, Gething PW, et al. A micro-epidemiological analysis of febrile malaria in Coastal Kenya showing hotspots within hotspots. eLife. 2014;3: e02130. doi: 10.7554/eLife.02130 24843017 PMC3999589

[7] Bousema T, Drakeley C, Gesase S, Hashim R, Magesa S, Mosha F, et al. Identification of hot spots of malaria transmission for targeted malaria control. J Infect Dis. 2010;201: 1764–1774. doi: 10.1086/652456 20415536

[8] Rouamba T, Nakanabo-Diallo S, Derra K, Rouamba E, Kazienga A, Inoue Y, et al. Socioeconomic and environmental factors associated with malaria hotspots in the Nanoro demographic surveillance area, Burkina Faso. BMC Public Health. 2019;19: 249. doi: 10.1186/s12889-019-6565-z 30819132 PMC6396465

[9] Ateba FF, Sagara I, Sogoba N, Touré M, Konaté D, Diawara SI, et al. Spatio-Temporal Dynamic of Malaria Incidence: A Comparison of Two Ecological Zones in Mali. Int J Environ Res Public Health. 2020;17: E4698. doi: 10.3390/ijerph17134698 32629876 PMC7370019

[10] Saugeon C, Baldet T, Akogbeto M, Henry MC. [Will climate and demography have a major impact on malaria in sub-Saharan Africa in the next 20 years?]. Med Trop Rev Corps Sante Colon. 2009;69: 203–207. 19545045

[11] Nourein AB, Abass MA, Nugud AHD, Hassan IE, Snow RW, Noor AM. Identifying Residual Foci of Plasmodium falciparum Infections for Malaria Elimination: The Urban Context of Khartoum, Sudan. PLOS ONE. 2011;6: e16948. doi: 10.1371/journal.pone.0016948 21373202 PMC3044149

[12] Dieng S, Ba EH, Cissé B, Sallah K, Guindo A, Ouedraogo B, et al. Spatio-temporal variation of malaria hotspots in Central Senegal, 2008–2012. BMC Infect Dis. 2020;20: 424. doi: 10.1186/s12879-020-05145-w 32552759 PMC7301493

[13] Cissoko M, Sagara I, Sankaré MH, Dieng S, Guindo A, Doumbia Z, et al. Geo-Epidemiology of Malaria at the Health Area Level, Dire Health District, Mali, 2013–2017. Int J Environ Res Public Health. 2020;17: 3982. doi: 10.3390/ijerph17113982 32512740 PMC7312793

[14] Cissoko M, Magassa M, Sanogo V, Ouologuem A, Sangaré L, Diarra M, et al. Stratification at the health district level for targeting malaria control interventions in Mali. Sci Rep. 2022;12: 8271. doi: 10.1038/s41598-022-11974-3 35585101 PMC9117674

[15] Cissoko M, Sagara I, Landier J, Guindo A, Sanogo V, Coulibaly OY, et al. Sub-national tailoring of seasonal malaria chemoprevention in Mali based on malaria surveillance and rainfall data. Parasit Vectors. 2022;15: 278. doi: 10.1186/s13071-022-05379-4 35927679 PMC9351140

[16] Ouédraogo M, Kangoye DT, Samadoulougou S, Rouamba T, Donnen P, Kirakoya-Samadoulougou F. Malaria Case Fatality Rate among Children under Five in Burkina Faso: An Assessment of the Spatiotemporal Trends Following the Implementation of Control Programs. Int J Environ Res Public Health. 2020;17: 1840. doi: 10.3390/ijerph17061840 32178354 PMC7143776

[17] Samadoulougou S, Maheu-Giroux M, Kirakoya-Samadoulougou F, De Keukeleire M, Castro MC, Robert A. Multilevel and geo-statistical modeling of malaria risk in children of Burkina Faso. Parasit Vectors. 2014;7: 350. doi: 10.1186/1756-3305-7-350 25074132 PMC4262087

[18] Valle D, Millar J, Amratia P. Spatial heterogeneity can undermine the effectiveness of country-wide test and treat policy for malaria: a case study from Burkina Faso. Malar J. 2016;15: 513. doi: 10.1186/s12936-016-1565-2 27760546 PMC5070201

[19] Ouedraogo B, Inoue Y, Kambiré A, Sallah K, Dieng S, Tine R, et al. Spatio-temporal dynamic of malaria in Ouagadougou, Burkina Faso, 2011–2015. Malar J. 2018;17: 138. doi: 10.1186/s12936-018-2280-y 29609606 PMC5879937

[20] Sangaré I, Ouattara CA, Soma DD, Soma D, Assogba BS, Namountougou M, et al. Spatial-temporal pattern of malaria in Burkina Faso from 2013 to 2020. Parasite Epidemiol Control. 2022;18: e00261. doi: 10.1016/j.parepi.2022.e00261 35859938 PMC9289732

[21] Banque Mondiale. Population, total—Burkina Faso | Data]. [cited 2022 Jan 2]. Available from: https://donnees.banquemondiale.org/indicator/SP.POP.TOTL?locations=BF

[22] World Health Organization AFRO. Profil sanitaire du Burkina. [cited 2022 Jan 2]. Available from: https://www.afro.who.int/sites/default/files/2018-08/Profil%20sanitaire%20du%20Burkina%20%202.pdf

[23] World Health Organization. Disease surveillance for malaria elimination: an operational manual. Surveill Épidémiologique En Vue Lélimination Palud Man Opérationnel. 2012 [cited 22 Mar 2023]. Available: https://apps.who.int/iris/handle/10665/44852

[24] Gorelick N, Hancher M, Dixon M, Ilyushchenko S, Thau D, Moore R. Google Earth Engine: Planetary-scale geospatial analysis for everyone. Remote Sens Environ. 2017;202: 18–27. doi: 10.1016/j.rse.2017.06.031

[25] Geofabrik Download Server. [cited 2 Jan 2022]. Available: https://download.geofabrik.de/africa/burkina-faso.html

[26] Friedman J, Tibshirani R. The Monotone Smoothing of Scatterplots. Technometrics. 1984;26: 243–250. doi: 10.1080/00401706.1984.10487961

[27] Ferreira CS, Zeller CB, Mimura AMS, Silva JCJ. Partially linear models and their applications to change point detection of chemical process data. J Appl Stat. 2017;44: 2125–2141. doi: 10.1080/02664763.2016.1247788

[28] Optimal Detection of Changepoints With a Linear Computational Cost: Journal of the American Statistical Association: Vol 107, No 500. [cited 3 Jan 2022]. Available: https://www.tandfonline.com/doi/abs/10.1080/01621459.2012.737745

[29] Killick R, Eckley IA. changepoint: An R Package for Changepoint Analysis. J Stat Softw. 2014;58: 1–19. doi: 10.18637/jss.v058.i03

[30] Zhang NR, Siegmund DO. A modified Bayes information criterion with applications to the analysis of comparative genomic hybridization data. Biometrics. 2007;63: 22–32. doi: 10.1111/j.1541-0420.2006.00662.x 17447926

[31] Sharma S, Swayne DA, Obimbo C. Trend analysis and change point techniques: a survey. Energy Ecol Environ. 2016;1: 123–130. doi: 10.1007/s40974-016-0011-1

[32] Arif SNAM, Mohsin MFM, Bakar AA, Hamdan AR, Abdullah SMS. CHANGE POINT ANALYSIS: A STATISTICAL APPROACH TO DETECT POTENTIAL ABRUPT CHANGE. J Teknol. 2017;79. doi: 10.11113/jt.v79.10388

[33] Wilke CO. ggridges: Ridgeline Plots in “ggplot2.” 2021. Available: https://CRAN.R-project.org/package=ggridges

[34] Kulldorff M. A spatial scan statistic. Commun Stat—Theory Methods. 1997;26: 1481–1496. doi: 10.1080/03610929708831995

[35] Kleinman K (2015). _rsatscan: Tools, Classes, and Methods for Interfacing with SaTScan Stand-Alone Software_. R package version 0.3.9200, <http://www.satscan.org>.

[36] Kimuyu JS, Muthama NJ, Musyoka SM. Ecological Niche Modeling For Spatial-Temporal Quantification Of The Changing Dynamics Of Malaria Vector Distribution In Kenya Under Climate Change Forcing. 2017;6: 14.

[37] Martiny N, Dessay N, Yaka P, Toure O, Sultan B, Rebaudet S, et al. Le climat, un facteur de risque pour la santé en Afrique de l’Ouest. La Météorologie. 2012; 73–79. doi: 10.4267/2042/48135

[38] Sohil F, Sohali MU, Shabbir J. An introduction to statistical learning with applications in R: by Gareth James, Daniela Witten, Trevor Hastie, and Robert Tibshirani, New York, Springer Science and Business Media, 2013, $41.98, eISBN: 978-1-4614-7137-7. Stat Theory Relat Fields. 2021; 1–1. doi: 10.1080/24754269.2021.1980261

[39] Jolliffe IT. PRINCIPAL COMPONENT ANALYSIS: A BEGINNER’S GUIDE—I. Introduction and application. Weather. 1990;45: 375–382. doi: 10.1002/j.1477-8696.1990.tb05558.x

[40] Rucker C, Buchet L. Distinction, par les micro- striations dentaires, d’individus issus d’environnements et d’horizons chronologiques différents: Apport de l’analyse factorielle et de la classification ascendante hiérarchique/Advantages of the utilization of factorial analysis of correspondences and hierarchical ascendant classification for the distinction, based on dental micro-striations, of individuals originating from different chronological horizons. Paléo Rev Archéologie Préhistorique. 1998;10: 7–16. doi: 10.3406/pal.1998.1125

[41] Abdesselam R. A Topological Approach of Principal Component Analysis. Int J Data Sci Anal. 2021;77. doi: 10.11648/j.ijdsa.20210702.11

[42] Murtagh F, Legendre P. Ward’s Hierarchical Agglomerative Clustering Method: Which Algorithms Implement Ward’s Criterion? J Classif. 2014;31: 274–295. doi: 10.1007/s00357-014-9161-z

[43] Murtagh F, Legendre P. Ward’s Hierarchical Clustering Method: Clustering Criterion and Agglomerative Algorithm. J Classif. 2014;31: 274–295. doi: 10.1007/s00357-014-9161-z

[44] Generalized Additive Models | T.J. Hastie, R.J. Tibshirani | Taylor &. [cited 3 Jan 2022]. Available: https://www.taylorfrancis.com/books/mono/10.1201/9780203753781/generalized-additive-models-hastie-tibshirani

[45] Guisan A, Edwards TC, Hastie T. Generalized linear and generalized additive models in studies of species distributions: setting the scene. Ecol Model. 2002;157: 89–100. doi: 10.1016/S0304-3800(02)00204-1

[46] Wood SN. Generalized Additive Models: an introduction with R.: 397.

[47] Sim CH. First-order autoregressive models for gamma and exponential processes. J Appl Probab. 1990;27: 325–332. doi: 10.2307/3214651

[48] Al-Osh MA, Aly E-EAA. First order autoregressive time series with negative binomial and geometric marginals. Commun Stat—Theory Methods. 1992;21: 2483–2492. doi: 10.1080/03610929208830925

[49] Guo C, Yang L, Ou C-Q, Li L, Zhuang Y, Yang J, et al. Malaria incidence from 2005–2013 and its associations with meteorological factors in Guangdong, China. Malar J. 2015;14: 116. doi: 10.1186/s12936-015-0630-6 25881185 PMC4389306

[50] Xiang J, Hansen A, Liu Q, Tong MX, Liu X, Sun Y, et al. Association between malaria incidence and meteorological factors: a multi-location study in China, 2005–2012. Epidemiol Infect. 2018;146: 89–99. doi: 10.1017/S0950268817002254 29248024 PMC9134552

[51] Gu C, Wahba G. Minimizing GCV/GML Scores with Multiple Smoothing Parameters via the Newton Method. SIAM J Sci Stat Comput. 1991;12: 383–398. doi: 10.1137/0912021

[52] Cai W. 378–2008: Fitting Generalized Additive Models with the GAM Procedure in SAS® 9.2. 2008; 14.

[53] Croissance de la population en Burkina Faso. In: DonnéesMondiales.com [Internet]. [cited 3 Apr 2023]. Available: https://www.donneesmondiales.com/afrique/burkina-faso/croissance-population.php

[54] Martineau P, Behera SK, Nonaka M, Jayanthi R, Ikeda T, Minakawa N, et al. Predicting malaria outbreaks from sea surface temperature variability up to 9 months ahead in Limpopo, South Africa, using machine learning. Front Public Health. 2022;10. Available: https://www.frontiersin.org/articles/10.3389/fpubh.2022.962377" xlink:type="simple">https://www.frontiersin.org/articles/10.3389/fpubh.2022.962377 36091554 10.3389/fpubh.2022.962377 PMC9453600

[55] Spatio-temporal analysis and prediction of malaria cases using remote sensing meteorological data in Diébougou health district, Burkina Faso, 20162017 | Scientific Reports. [cited 26 May 2022]. Available: https://www.nature.com/articles/s41598-021-99457-9 10.1038/s41598-021-99457-9 PMC8501026 34625589

[56] Tagbor H, Cairns M, Bojang K, Coulibaly SO, Kayentao K, Williams J, et al. A Non-Inferiority, Individually Randomized Trial of Intermittent Screening and Treatment versus Intermittent Preventive Treatment in the Control of Malaria in Pregnancy. PloS One. 2015;10: e0132247. doi: 10.1371/journal.pone.0132247 26258474 PMC4530893

[57] Dicko A, Diallo AI, Tembine I, Dicko Y, Dara N, Sidibe Y, et al. Intermittent preventive treatment of malaria provides substantial protection against malaria in children already protected by an insecticide-treated bednet in Mali: a randomised, double-blind, placebo-controlled trial. PLoS Med. 2011;8: e1000407. doi: 10.1371/journal.pmed.1000407 21304923 PMC3032550

[58] Malaria in Africa. In: UNICEF DATA [Internet]. [cited 15 Mar 2023]. Available: https://data.unicef.org/topic/child-health/malaria/

[59] Kangoye DT, Noor A, Midega J, Mwongeli J, Mkabili D, Mogeni P, et al. Malaria hotspots defined by clinical malaria, asymptomatic carriage, PCR and vector numbers in a low transmission area on the Kenyan Coast. Malar J. 2016;15: 213. doi: 10.1186/s12936-016-1260-3 27075879 PMC4831169

[60] Afrane YA, Klinkenberg E, Drechsel P, Owusu-Daaku K, Garms R, Kruppa T. Does irrigated urban agriculture influence the transmission of malaria in the city of Kumasi, Ghana? Acta Trop. 2004;89: 125–134. doi: 10.1016/j.actatropica.2003.06.001 14732235

[61] Dhiman RC, Pahwa S, Dhillon GPS, Dash AP. Climate change and threat of vector-borne diseases in India: are we prepared? Parasitol Res. 2010;106: 763–773. doi: 10.1007/s00436-010-1767-4 20155369

[62] Understanding the link between malaria risk and climate | PNAS. [cited 16 Feb 2023]. Available: https://www.pnas.org/doi/10.1073/pnas.0903423106

[63] Ceccato P, Ghebremeskel T, Jaiteh M, Graves PM, Levy M, Ghebreselassie S, et al. Malaria Stratification, Climate, and Epidemic Early Warning in Eritrea. Defining and Defeating the Intolerable Burden of Malaria III: Progress and Perspectives: Supplement to Volume 77(6) of American Journal of Tropical Medicine and Hygiene. American Society of Tropical Medicine and Hygiene; 2007. Available: https://www.ncbi.nlm.nih.gov/books/NBK1682/18165476

[64] Bayoh MN, Lindsay SW. Effect of temperature on the development of the aquatic stages of Anopheles gambiae sensu stricto (Diptera: Culicidae). Bull Entomol Res. 2003;93: 375–381. doi: 10.1079/ber2003259 14641976

[65] Lyons CL, Coetzee M, Chown SL. Stable and fluctuating temperature effects on the development rate and survival of two malaria vectors, Anopheles arabiensis and Anopheles funestus. Parasit Vectors. 2013;6: 104. doi: 10.1186/1756-3305-6-104 23590860 PMC3637585

[66] Beck-Johnson LM, Nelson WA, Paaijmans KP, Read AF, Thomas MB, Bjørnstad ON. The Effect of Temperature on Anopheles Mosquito Population Dynamics and the Potential for Malaria Transmission. PLoS ONE. 2013;8: e79276. doi: 10.1371/journal.pone.0079276 24244467 PMC3828393

[67] Paaijmans KP, Cator LJ, Thomas MB. Temperature-Dependent Pre-Bloodmeal Period and Temperature-Driven Asynchrony between Parasite Development and Mosquito Biting Rate Reduce Malaria Transmission Intensity. PLOS ONE. 2013;8: e55777. doi: 10.1371/journal.pone.0055777 23383280 PMC3561307

[68] Himeidan YE, Kweka EJ. Malaria in East African highlands during the past 30 years: impact of environmental changes. Front Physiol. 2012;3: 315. doi: 10.3389/fphys.2012.00315 22934065 PMC3429085

[69] Ermert V, Fink AH, Morse AP, Paeth H. The Impact of Regional Climate Change on Malaria Risk due to Greenhouse Forcing and Land-Use Changes in Tropical Africa. Environ Health Perspect. 2012;120: 77–84. doi: 10.1289/ehp.1103681 21900078 PMC3261943

[70] Coulibaly D, Rebaudet S, Travassos M, Tolo Y, Laurens M, Kone AK, et al. Spatio-temporal analysis of malaria within a transmission season in Bandiagara, Mali. Malar J. 2013;12: 82. doi: 10.1186/1475-2875-12-82 23452561 PMC3618208

