# Supplementary material for: Malaria in Burkina Faso: A comprehensive analysis of spatiotemporal distribution of incidence and environmental drivers, and implications for control strategies
Source: PLoS One. 2023 Sep 13;18(9):e0290233. doi: 10.1371/journal.pone.0290233 (PMC10499254; doi:10.1371/journal.pone.0290233)
Supplement: S1 Table — (DOCX) [file pone.0290233.s001.docx]

| **Variables** | **Sources** | **Resolution** |
| --- | --- | --- |
| daily precipitation | Climate Hazards Group InfraRed Precipitation with Station Data | 5.56 km |
| average daily temperature | Latest climate reanalysis produced by ECMWF / Copernicus Climate Change Service | 27.78 km |
| maximum daily temperature | Latest climate reanalysis produced by ECMWF / Copernicus Climate Change Service | 27.78 km |
| minimum daily temperature | Latest climate reanalysis produced by ECMWF / Copernicus Climate Change Service | 27.78 km |
| 8 days normalized difference vegetation index | MODIS Terra Surface Reflectance 8-Day Global | 0.5 km |
| daily surface pressure | Latest climate reanalysis produced by ECMWF / Copernicus Climate Change Service | 27.78 km |
| 8 days fire detection information | Fire Information for Resource Management System | 1 km |

**S1 Table. Meteorological and environmental variables**
