# Supplementary figures and images for: Malaria in Burkina Faso: A comprehensive analysis of spatiotemporal distribution of incidence and environmental drivers, and implications for control strategies
Source: PLoS One. 2023 Sep 13;18(9):e0290233. doi: 10.1371/journal.pone.0290233 (PMC10499254; doi:10.1371/journal.pone.0290233)

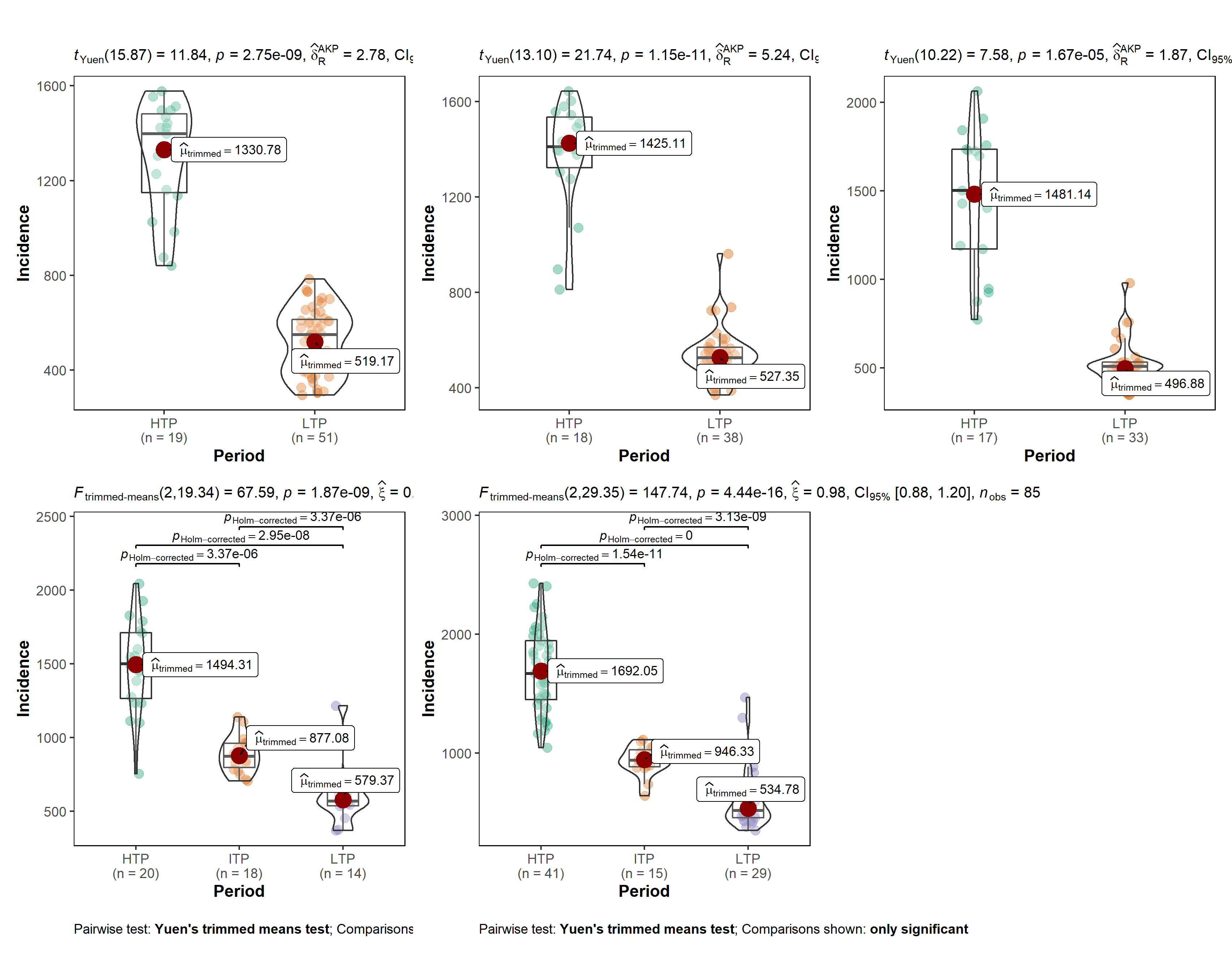

Supplement: S1 Fig — Difference in the mean of the incidence through epidemics transmission periods using the Yuen’s test for trimmed means (robust t-test) for two groups and the empirical likelihood-based ANOVA for trimmed means test for more than two groups. (TIF) [file pone.0290233.s002.tif]

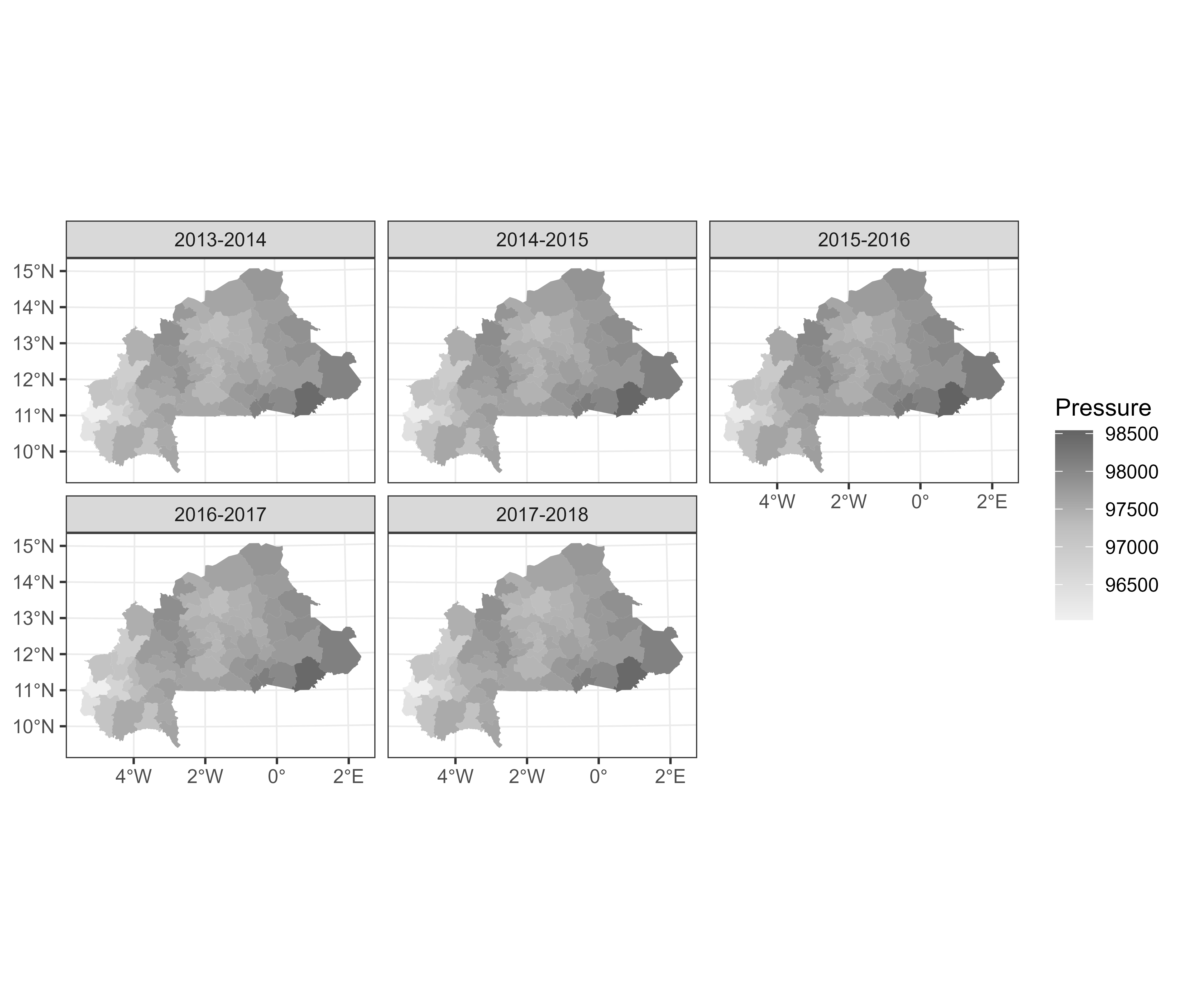

Supplement: S2 Fig — (TIF) [file pone.0290233.s003.tif]

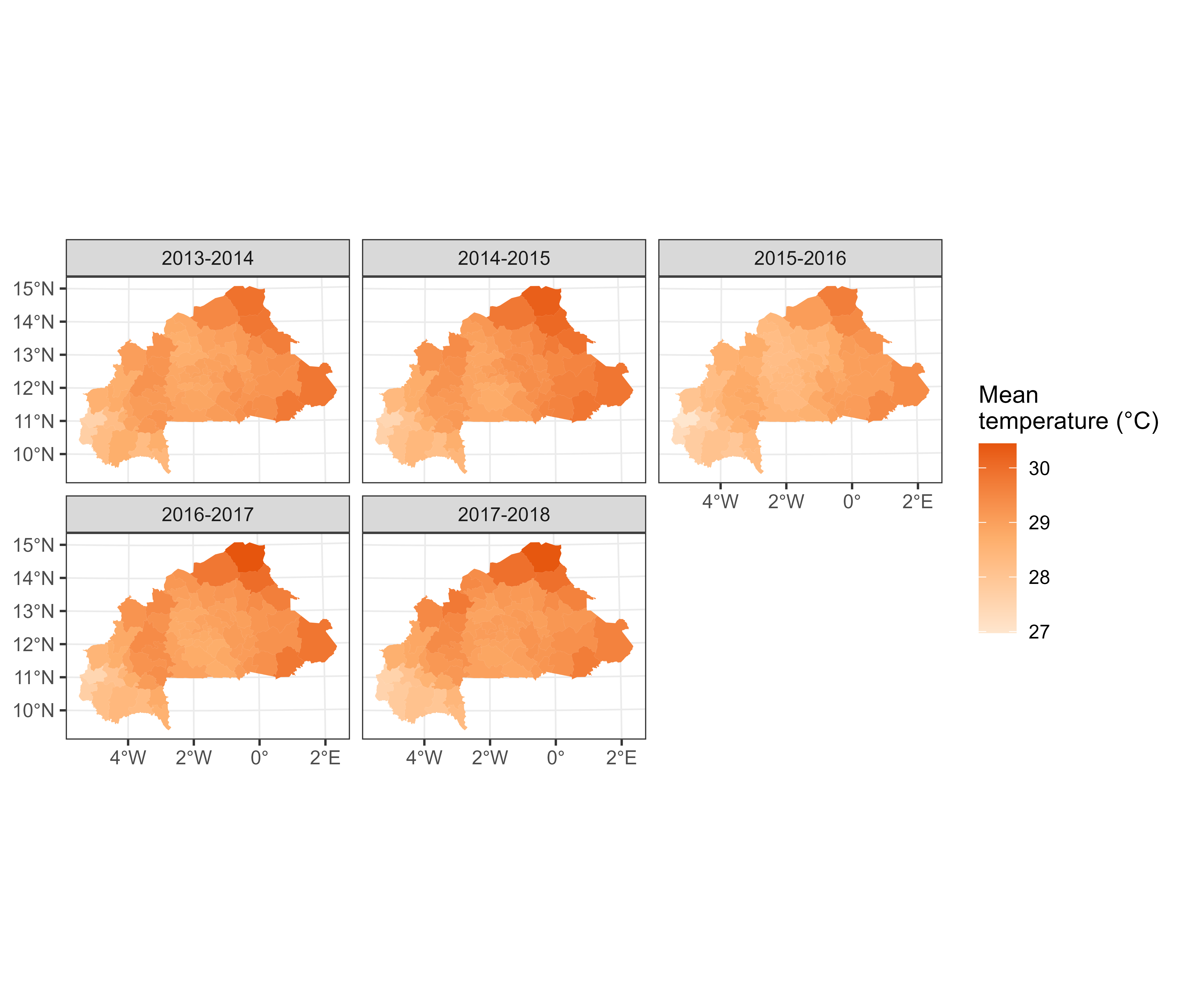

Supplement: S3 Fig — (TIF) [file pone.0290233.s004.tif]

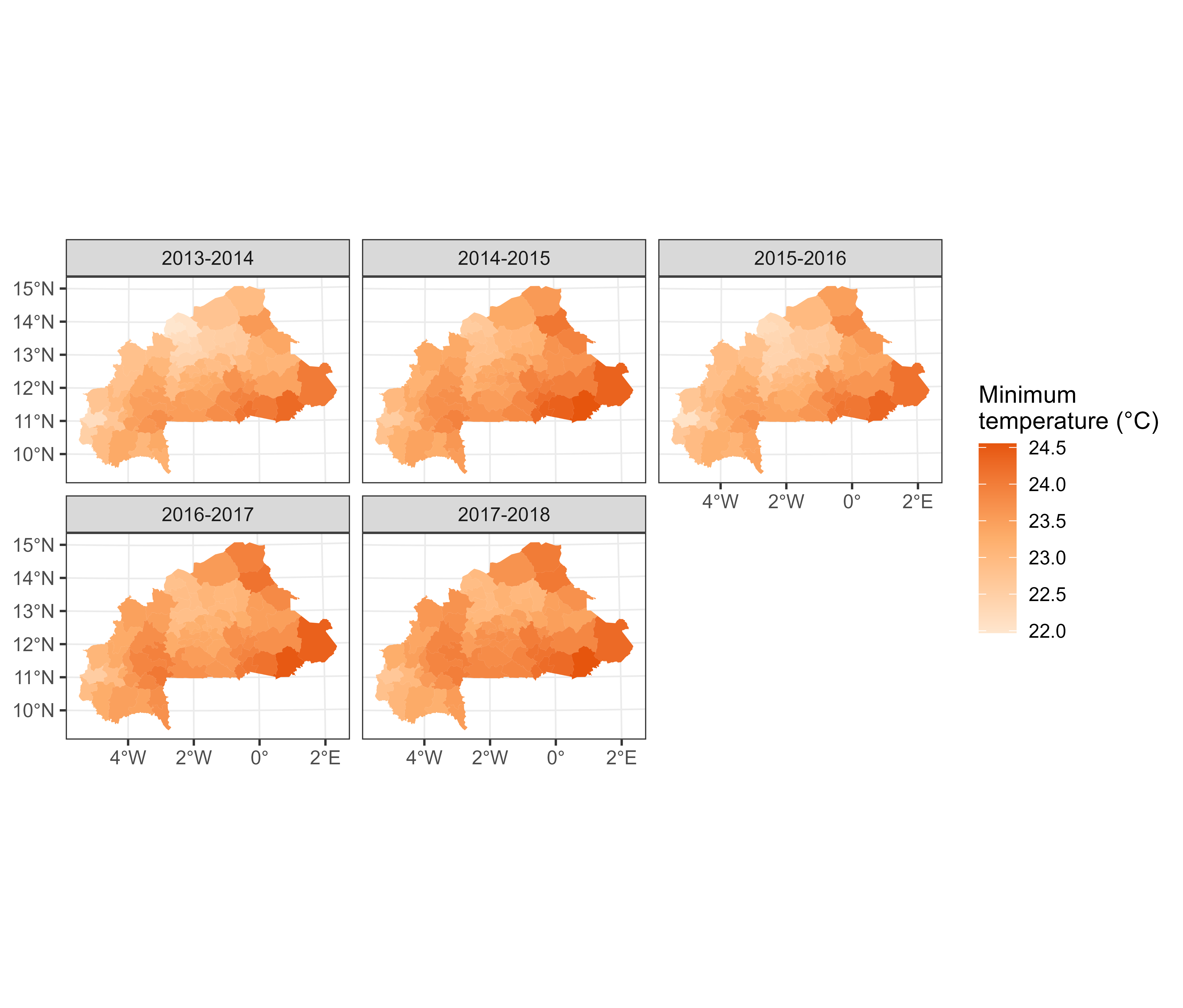

Supplement: S4 Fig — (TIF) [file pone.0290233.s005.tif]

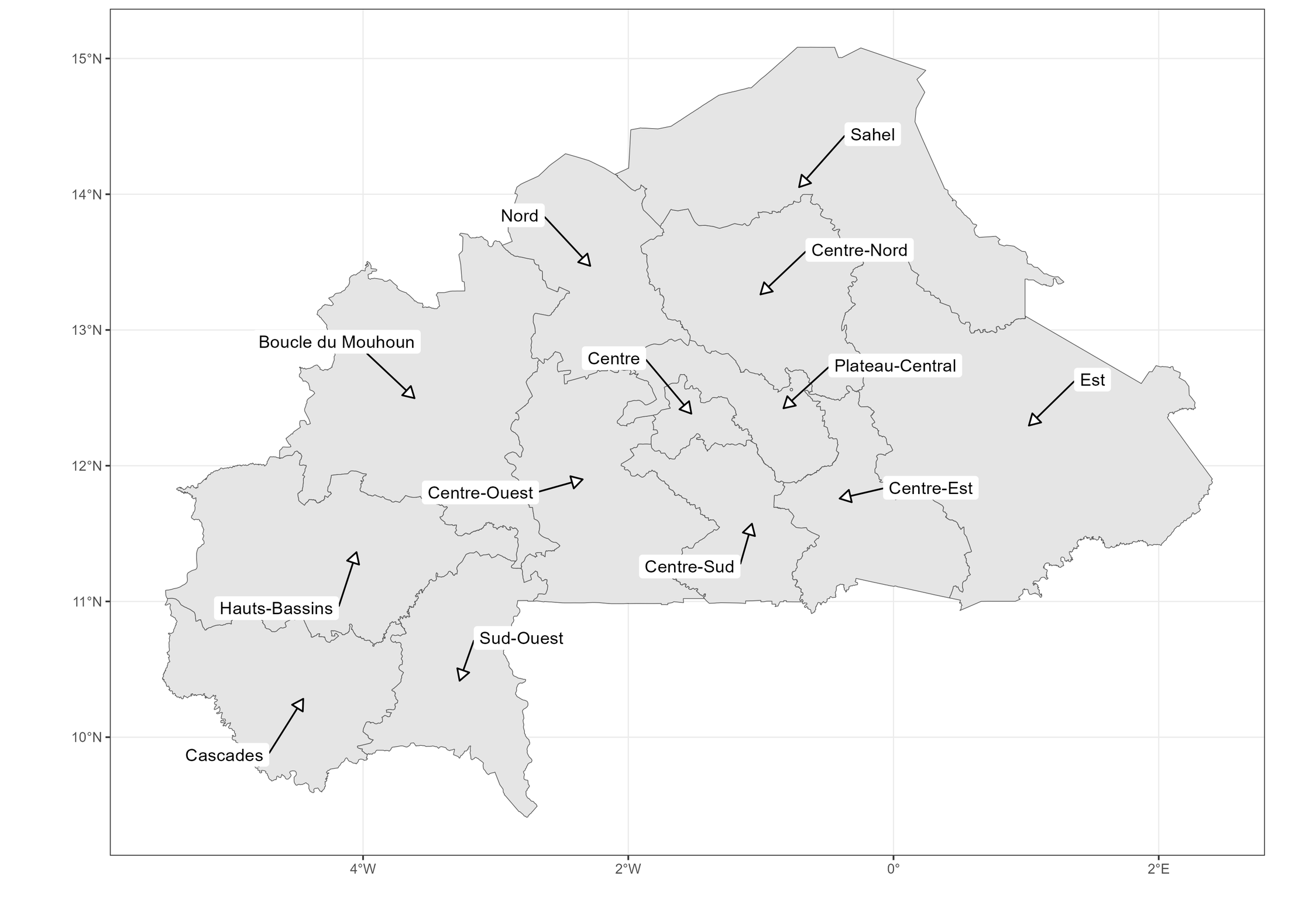

Supplement: S5 Fig — (TIF) [file pone.0290233.s006.tif]
